# Crystallochromism: A Hybrid Model for the Spectral Properties of Quinacridone Polymorphs

**DOI:** 10.1021/acs.jctc.5c01022

**Published:** 2025-10-29

**Authors:** Lorenzo Savi, Matteo Masino, Anna Painelli, Luca Grisanti

**Affiliations:** † Department of Chemistry, Life Science and Environmental Sustainability, 9370 Parma University, Parco Area delle Scienze 17/A, 43124 Parma, Italy; ‡ Division of Theoretical Physics, Ruder Bošković Institute, Bijenička cesta 54, 10000 Zagreb, Croatia; § 518735 CNR - Istituto Officina dei Materiali (IOM) c/o SISSA (International School for Advanced Studies), via Bonomea 256, 34136 Trieste, Italy

## Abstract

The evolution of the optical properties of a molecule from solution to a crystalline phase is nontrivial, as it results from a complex interplay of several interactions, including electrostatic and charge-transfer intermolecular interactions and the coupling with molecular vibrations. In order to address the crystallochromism observed in quinacridone (QA), a hybrid modeling strategy is presented that successfully describes the optical properties (absorption and emission) of the βQA and γQA crystalline phases. The proposed protocol relies on the parametrization of the Frenkel–Holstein Hamiltonian against quantum chemical calculations. Periodic density functional theory (DFT) is adopted to optimize the crystallographic geometry and to extract effective atomic charges. Time-dependent DFT (TD-DFT) results on the isolated molecule are exploited to parametrize the Holstein coupling, while TD-DFT results on the embedded molecules and on embedded clusters of increasing size are finally exploited to extract the exciton model parameters. For safe validation, the missing optical spectra of the two polymorphs were measured. The approach is general and paves the way for the rationalization of crystallochromism of molecular condensed phases.

## Graphical abstract

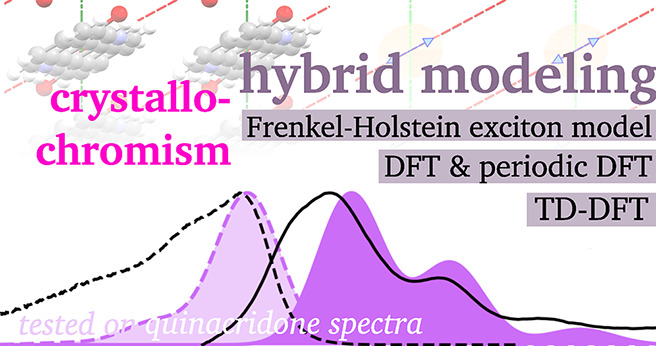

## 1 Introduction

The control and interpretation of the optical properties of functional molecular materials is a fundamental research topic in material science, with a wide range of applications in color industry,^1^ optoelectronic devices,^2,3^ fluorescent sensing,^4^ and bioimaging.^5^ The precisely engineered energy fluxes driven by intermolecular interactions in molecular assemblies are crucial to the photosynthetic processes and, if properly designed, can be exploited for quantum computing.^6,7^ A recent challenge in the field is the design of molecular luminophores for light generation and amplification.^8^ Luminescence, the radiative relaxation from excited states (fluorescence or phosphorescence), is rare in the solid state due to dominant nonradiative decay pathways.^9^ The introduction of aggregation-induced emission (AIE) in 2001 ^2,10^ pushed new possibilities for material design, fabrication, and device applications, particularly in achieving multicolor and white-light emission.^11^ Along these lines, several luminescent-related properties of condensed phases were discovered, including thermochromism,^12^ mechanochromism,^13,14^ and piezochromism.^15^ At a more fundamental stage, crystallochromism describes the property of a crystalline material to show different colors in the solid state. As with other more complex phenomena, crystallochromism relies on a subtle interplay of different interactions.

Here, we will focus on a specific form of crystallochromism, sometimes dubbed color polymorphism,^16^ that applies to systems showing different colors in different polymorphs. In these systems, the presence of specific interactions (such as hydrogen bonds, charge transfer, or π–π interactions) can give rise to new electronic transitions or even suppress transitions present at the molecular level. In this direction, crystal engineering is a helpful strategy to govern intermolecular interactions toward specific properties.^17^ In a broader perspective, crystallochromism sometimes refers to crystals where different optical spectra are due to different molecular configurations inside the crystal, caused by the presence of different conformers or rotamers building up the crystal. Crystallochromism finds several technological applications in temperature sensing,^18–20^ optoelectronic devices, and multiplex capabilities.^21^ Among the most studied examples of crystallochromic molecular systems, we mention *N*-(4-methyl-2-nitrophenyl)­acetamide,^22^ the ROY pigment,^23^ *N*-picryl-*p*-toluidine,^24^ and quinacridone (QA),^25^ which is the system tested in this work.

QA (5,12-Dihydroquinolino­[2,3-*b*]­acridine-7,14-dione) belongs to a well-known class of organic dyes and crystallochromic systems. QA, commonly known as “Pigment Violet 19,” has an intense violet color. The QA scaffold can be easily functionalized to tune its properties. Several QA derivatives are known, displaying different colors. For these reasons, QA and its derivatives are widely used in industry as organic pigments.^26–28^ QA is used in inks as the main product, but also in the sectors of digital printing, paints and coatings, plastics industry, textiles industry, and others.^29^ With its highly conjugated scaffold, QA has semiconducting properties: QA and its derivatives are extensively used for organic light-emitting devices (OLED),^30^ organic field effect transistor (OFET),^31^ organic solar cells (OSC)^32^, and other organic electronic devices. While its electronic structure and transport properties have been deeply investigated,^33–36^ less comprehensive studies deal with its optical properties.

The accurate prediction and simulation of the optical properties of molecular crystals remain a challenging theoretical and computational task. Excited states in molecular condensed phases are typically described as Frenkel excitons (FEs) to account for the delocalization of the molecular excitation as a result of electrostatic intermolecular interactions.^37^ Intermolecular charge-transfer (CT) excitations may also be involved, leading to a considerably more complex picture.^38–41^ Moreover, molecular vibrations have a large impact on spectral line shapes and therefore must be taken into account to properly simulate the color of the crystal.^42–44^

Model Hamiltonians proved useful to describe low-energy excited states in supramolecular systems, including molecular aggregates and crystals. Excited states and FEs can be described by means of a Frenkel–Holstein Hamiltonian accounting for electrostatic interactions among states with one excitation per molecular site and for electron-vibration coupling in the linear approximation.^44–46^ Essential-state models^47–49^ have been widely employed to describe CT transitions in donor–acceptor molecular systems, including aggregates.^39,40,50–56^ Essential-state Hamiltonians can be parametrized against experimental data^48,49^ and/or against results of quantum chemical calculations.^53,57^

At the electronic level, density functional theory (DFT) and its time-dependent DFT implementation (TD-DFT)^58^ have been largely and successfully applied to describe the excited states of isolated molecular systems, provided a proper functional is selected. Some critical aspects of DFT when dealing with organic conjugated systems,^59^ have recently been mitigated by a number of improvements, with double hybrid^60^ and long-range corrected functionals.^59,61^ However, the simulation of spectral properties in a crystalline environment remains hardly approachable with first-principles electronic structure calculations. With few exceptions,^62^ the simulation of emission with periodic ab initio methods is not available in any electronic structure codes and, therefore, is hardly applied in systematic approaches. More generally, addressing optical properties of molecular crystals requires several approximations, in the flavor of multimethods, such as for spectral warping^63^ or approximated Becke’s virial exciton model to entirely bypass conventional excited-state methods.^64^ A strategy that combines DFT with classical embedding (QM:MM) was proposed by Adamo et al.,^65,66^ and applied to model luminescence of various molecular crystals. Finally, the Crespo-Otero group proposed a cluster-based QM:QM′ protocol for electrostatic embedding, useful not only for optical properties but also for excited-state dynamics and photochemistry.^67^ Lately, they have been benchmarking several approaches for embedding.^68^

To step in a different direction and overcome some of the limitations of DFT, we propose here a hybrid modeling strategy that combines first-principles calculations and a model Hamiltonian. Specifically, we run TD-DFT calculations on isolated molecules (monomers) and molecular clusters derived from the crystal structure. These small crystal fragments are electrostatically embedded in a large portion of the crystal, following the charge distribution evaluated with periodic DFT. Relevant results are then exploited to parametrize a periodic model Hamiltonian that describes FE in the presence of electron-vibration coupling (Frenkel–Holstein), fully accounting for the excitonic dispersion in different directions.

In the FE model, the proper definition of the exciton coupling *J* is crucial. It measures the electrostatic interaction between electronic excitations on different molecules, and several strategies have been proposed for its estimation. The simplest approach relies on the point-dipole approximation. More sophisticated strategies have been developed, including (cube) transition densities^69^ and approaches based on dimer adiabatic excited states,^70^ successfully employed by some of us to describe triplet exciton couplings.^71^ Time-dependent tight-binding-based DFT was also attempted to go beyond the point-dipole approximation.^72^ In this work, we propose an original strategy inspired by the reverse eigenvalue problem. Specifically, the exciton couplings relevant to a crystal fragment are adjusted to reproduce the excitation energies and oscillator strengths obtained from TD-DFT calculations on molecular clusters of increasing size. The proposed general workflow has a modular structure applicable to different molecular crystals. The method is validated against absorption and fluorescence spectra of two QA polymorphs. Reliable fluorescence data are available on films,^73^ but information on absorption spectra is very limited.^28^ To fill this gap, we collected experimental absorption and fluorescence spectra of the two polymorphs.

In the next section, we introduce the general theoretical methodological workflow and provide details about computational and experimental techniques. In the Results and Discussion, the approach is applied to the QA polymorphs, and calculated spectra are compared with experimental data.

## 2 Methods

To model the optical properties of QA, a hybrid modeling approach with a modular structure is proposed, as illustrated in Figure 1. First-principle calculations are exploited to evaluate the parameters to be plugged into an effective Frenkel–Holstein Hamiltonian that accounts for exciton delocalization and vibrational degrees of freedom in a linear coupling approach.

**Figure 1:**
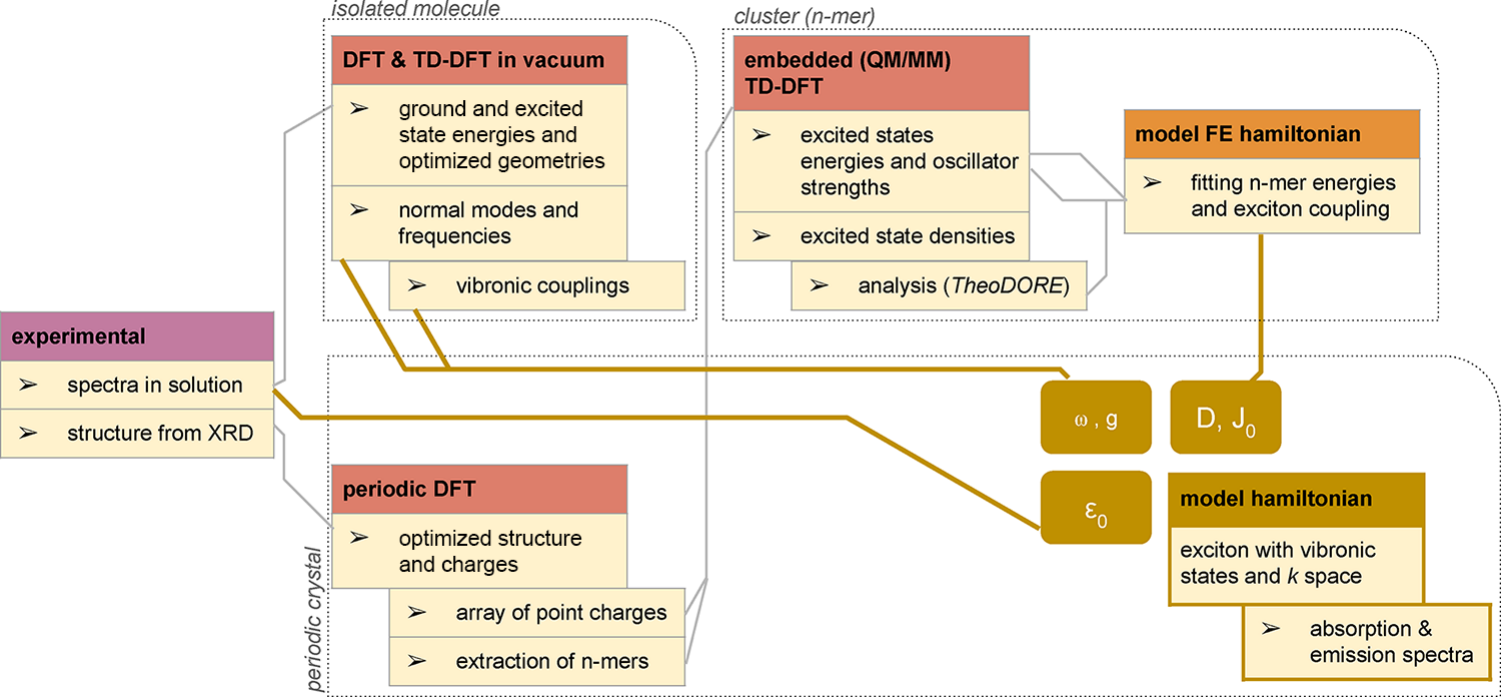
A schematic representation of the hybrid computational methodology.

### 2.1 Model Selection

The structures of βQA and γQA are taken from the literature.^25^ In both phases, quasi-independent 2D layers are found. Figure 2 offers a schematic representation of the structures and of the main intermolecular interactions. A more detailed description can be found in the electronic Supporting Information (ESI) Figures S1 and S2.

**Figure 2:**
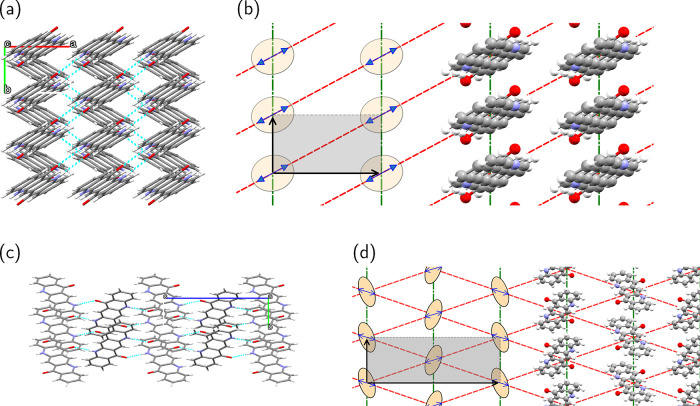
(a, b) βQA crystal structure. (a) βQA viewed from the *ab* plane. (b) Schematic representation of relevant interactions in βQA. (c, d) γQA crystal structure. (c) γQA viewed from the *bc* plane. (d) Schematic representation of relevant interactions in γQA. In panels (b) and (d), the gray box shows the unit cell, green lines mark π–π stacking interactions, red lines mark the hydrogen–bond interactions, and blue arrows are aligned with the molecular transition dipole moment.

### 2.2 Electronic Structure Calculations (Periodic DFT)

Periodic DFT (pDFT) calculations were performed using Quantum Espresso (QE) v 6.8 with vdw-df-cx functional^74^ to accurately establish the atomic positions and the electrostatic landscape (electron density), fully accounting for periodicity. GBRV ultrasoft PBE pseudopotentials are employed with fixed electronic occupation for the wave function with a grid of 5 × 10 × 2 = 100 *K*-points. Kinetic-energy cutoffs of 60 Ry for the wave function and of 600 Ry for the charge density were chosen. Default QE options were adopted, but with a tighter threshold for SCF convergence (10^–8^ a.u.). Initial coordinates from experimental CCDC structures βQA: QNACRD07 (no. 620258) and γQA: QNACRD07 (# 620259) were relaxed according to the BFGS algorithm with variable cell.

### 2.3 Excited States (TD-DFT)

Starting from the optimized periodic structures, crystalline fragments (monomer, dimer, and 1D molecular clusters) were selected across preferential interaction directions, e.g., along the π stacking direction or the hydrogen-bond direction. TD-DFT calculations with a QM/MM embedding (vide infra) were run to estimate transition energies and dipole moments on the embedded crystalline fragments. The ωB97X-D3BJ^75^ functional with the def2-TZVP basis set^76^ was selected, as available in Orca^77^ 5, that actually corresponds to the 10-parameter ωB97X-V^78^ functional with dispersion corrections (D3BJ version). The choice of the functional for the DFT part of our workflow is motivated by the good balance between the accuracy and computational cost achieved by range-separated DFT functionals. Their performance was confirmed by recent benchmark studies.^79–81^ For these Gaussian-based DFT calculations, we employed the Orca 5.0.2 package,^77^ (by default), taking advantage of the resolution of identity approximation and of the Tamm–Dancoff approximation, TDA (for TD-DFT).^82^ TDA was adopted to achieve convergence and good stability of excited state solutions, as well as to reduce the computational burden for large clusters. The analysis of the nature of excited states was performed using the TheoDORE 3.0 package.^83–86^ Of special interest is the calculation of the electron–hole correlation Ω matrix defined over a set of fragments, where each Ω­(*A*, *B*) element quantifies the degree of electronic transfer associated with a given electronic transition when the hole is restricted to a fragment A of the system and the electron to fragment B. In other words, Ω matrices map the transfer of electronic population between the occupied *A* (*x*-axis) and virtual states *B* (*y*-axis) according to the predefined partition into fragments.^86^ Upon subdividing the system of interest into fragments, electron–hole correlation plots are reported in terms of such Ω matrices. From this analysis, reliable information is retrieved on the nature of excited states, including their CT character and the exciton delocalization.

### 2.4 Electrostatic Embedding

The QA molecule or QA clusters defined above were inserted into a large grid of point charges to effectively create a QM/MM-like crystalline embedding (in-house code). Specifically, atomic point charges obtained via a Löwdin analysis from the pDFT calculations were located at the atomic positions. Figure S5 in ESI illustrates the impact of embedding a monomer in successive layers of charges (for the central monomer, one layer corresponds to 3^3^–1 = 26 molecules of point charge arrays, 2 layers correspond to 5^3^–1 = 124, etc.). Acceptable convergence is reached at 3 layers, i.e., introducing 26 unit cells with point-charges around the DFT unit cell.

**Figure 3:**
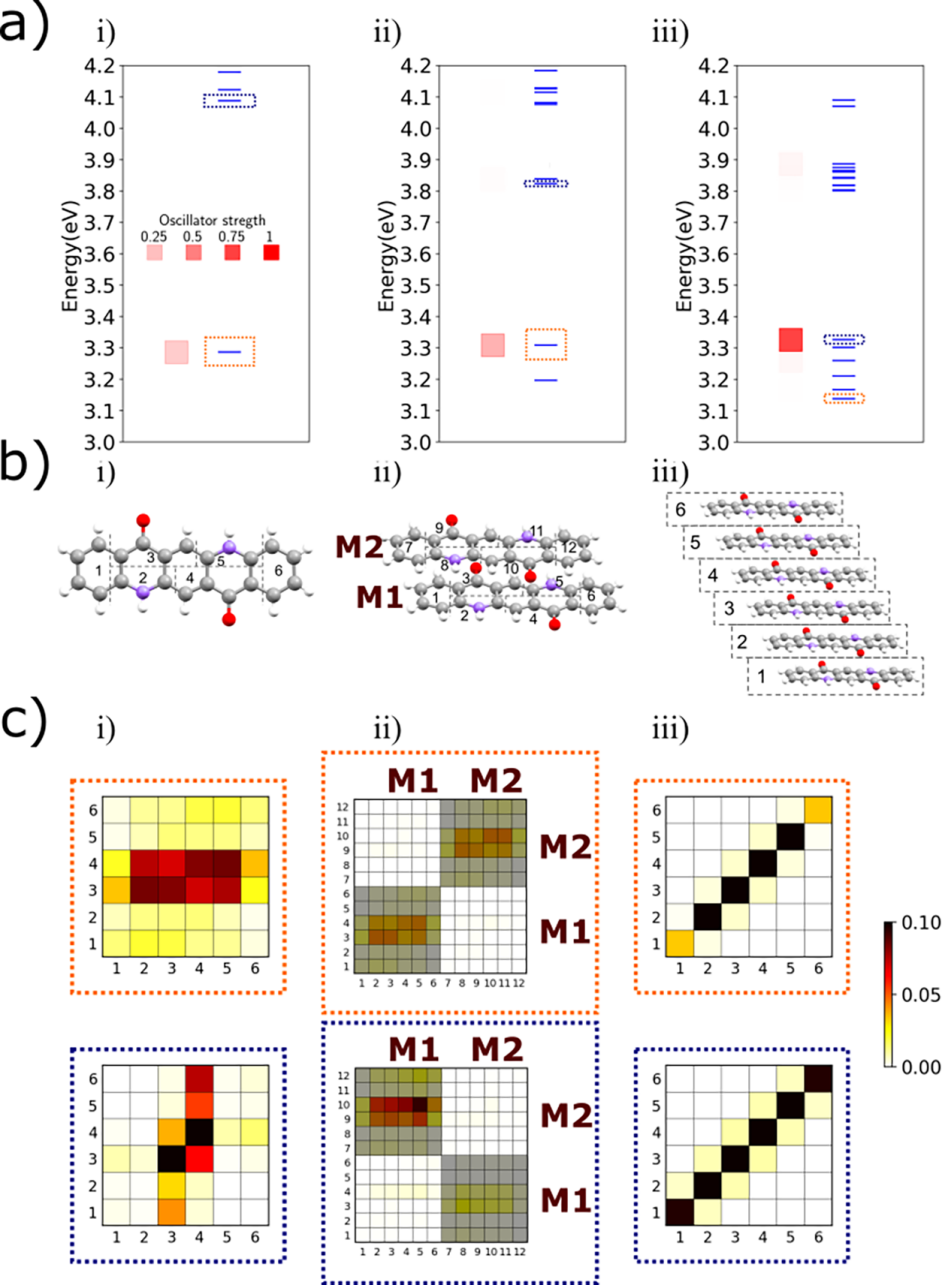
Building the exciton model for QA from monomer to βQA hexamer. (a) Transition energy diagrams: blue bars representing the TD-DFT excitation energies of the embedded monomer (i), dimer (ii), and hexamer (iii). The color intensity of the red square represents the oscillator strength according to the scale in panel a­(i). The blue and orange dotted frames in panels (i–iii) mark the states represented in panels c (i–iii). (b) The molecular structure of the monomer (i), the dimer (ii), and the hexamer (iii), and their fragment subdivision for this analysis. M1 and M2 are shown for the dimers to help interpret molecular vs fragment information. (c) Electron–hole correlation analysis of selected transitions for the monomer (i), dimer (ii), and hexamer (iii), carried out by TheoDORE version 3.0 (Ω matrices). The colorbar represents the degree of transferred electronic population.

### 2.5 Exciton Model Parameters

To estimate the exciton coupling strength, we adopt an inverse-eigenvalue problem approach. Specifically, we focus on embedded 1D clusters of *N* molecules, as extracted from the relaxed crystalline structure. In the hypothesis of equivalent molecular sites and only accounting for nearest neighbor interactions, the exciton Hamiltonian reads:

H^exc=ϵ∑iN|i⟩⟨i|+J∑iN−1(|i+1⟩⟨i|+|i⟩⟨i+1|) 1

where *i* runs on the *N* molecular sites, |*i*⟩ defines the state where the exciton resides on site *i*, ϵ is the excitation energy, and *J* is the site-independent exciton interaction energy. For each embedded hexamer, the TD-DFT (ωB97X, def2-TZVP basis set—see above) transition energies of the first excitonic manifold (a set of six excited states) are fitted by tuning the coupling and the diagonal energies of the exciton model, the sign of *J* being defined by the distribution of the TD-DFT oscillator strength. Notably, although not fitted, the distribution of oscillator strengths of the different eigenstates is well reproduced by the model. The electron–hole correlation analysis described above confirms the excitonic character of TD-DFT states, and in one case, spurious excitations caused by finite-size effects were removed. More details about the procedure are presented in the ESI Section 3, Figures S16, and S17.

The analysis is performed on 1D clusters developing along different directions, getting information on relevant exciton interactions. The exciton interaction energies obtained from the analysis of TD-DFT results account for the presence of fixed atomic charges in the surrounding crystal but not for the polarizability of the crystal. To partially correct for this, the *J* values extracted from the analysis of TD-DFT results are divided by the squared refractive index of the crystal to obtain the effective *J*_H,π_ to be inserted in the exciton model (eqs 4 and 5). The refractive indices of the two polymorphs are η = 2.04 for γQA and η = 2.02 for βQA.^87^

In principle, the fit of TD-DFT data with the Hamiltonian in eq 1 would also lead to an estimate of the on-site energies, ϵ_0_. However, as a common practice, TD-DFT calculated spectra are rigidly shifted to improve the agreement with the experiment. Here, we prefer a different strategy. QA, a symmetric nonpolar dye, is marginally solvatochromic, and the position of the maximum of its absorption band in solution offers a good estimate of ϵ_0_, the transition energy of an isolated QA in a polarizable environment. Fitting the absorption spectrum of QA in Dioxane,^88^ we estimate ϵ_0_ = 2.43 eV (see in ESI). The on-site energies entering eq 1 must also account for the shift of the transition energies of the molecule caused by the presence of the surrounding crystals. Accordingly, we set ϵ = ϵ_0_ + Δ, where Δ is the difference between the TD-DFT lowest excitation energy of the embedded and gas phase monomer.

### 2.6 DFT-Based Vibrational and Vibronic Properties

Vibrational frequencies, ω_*i*_, and Huang–Rhys factors, *S*_*i*_, are obtained from TD-DFT calculation on a single isolated monomer using the Orca package, employing the adiabatic Hessian after a step (AHAS) approximation, where a single optimization step is done in the excited-state geometry and then the Hessian is recalculated in that geometry.^89^ With this information, a single effective coupled vibrational mode is defined with a frequency that is the weighted average of the vibrational frequencies:^90,91^

$$\omega_{\mathrm{eff}}=\frac{\sum_{i}S_{i}\omega_{i}}{\sum_{i}S_{i}}$$ 2

and a relaxation energy that is the sum of all relaxation energies, λ = ∑_*i*_λ_*i*_ (with λ_*i*_ = *S*_*i*_ω_*i*_). Accordingly, the coupling constant of the effective coupled mode is

$$g=\sqrt{\omega_{\mathrm{eff}}\lambda }$$ 3

In this approach, the (typically minor) effect of the crystalline environment on intramolecular vibrational modes is neglected.

### 2.7 Frenkel–Holstein Model Hamiltonian

We employed a classical Frenkel–Holstein instrument (see Results and eq 6), parametrized according to the protocol described above. It is convenient to exploit the translational symmetry and rewrite the 2D periodic Hamiltonian in the reciprocal space. For the βQA phase with a single molecule per unit cell, the π-stacking interaction develops along the **b** axis, while H-bonds develop along the **a** + **b** direction, and the Hamiltonian in the reciprocal space reads:

H^β=∑k[ϵ0+D+2Jπβcos(kb)+2JHβcos(k(a+b))]b̃k†b̃k+hωeff∑q(ãq†ãq+12)+gN∑k,q[ãq†b̃k†b̃k+q+ã−q†b̃k†b̃k+q] 4

where *k* and *q* are the electronic and vibrational wavevectors, respectively, *a*_*i*_^†^ and *a*_*i*_ are the vibrational creation and annihilation operators for the effective vibrational mode on the *i*th molecule, *b*_*i*_^†^ and *b*_*i*_ are the creation and annihilation operators for the electronic excitation, *g* is the vibronic coupling constant, 2*J*_H_^β^ and *J*_π_^β^ define the two excitonic couplings between nearest neighbors. Further details are given in ESI, Section 4.1. For γQA, the transformed Hamiltonian is more complex due to the presence of two molecules per unit cell. Following the derivation in ESI, Section 4.2, we get:

H^γ=∑k{{ϵ0+D+2Jπγcos(kb)+2JHγcos[k(a+b2)]+2JHγcos[k(a−b2)]}b̃1,k†b̃1,k+{ϵ0+D+2Jπγcos(kb)−2JHγcos[k(a+b2)]−2JHγcos[k(a−b2)]}b̃2,k†b̃2,k}+hωeff∑q(ã1,q†ã1,q+ã2,q†ã2,q)+g2N∑k,q{ã1,q†b̃1,k†b̃1,k+q+ã1,q†b̃2,k†b̃2,k+q+ã2,q†b̃1,k†b̃2,k+q+ã2,q†b̃2,k†b̃1,k+q+h.c.} 5

where the same notation as in eq 4 is employed.

### 2.8 Simulated Spectra

The Frenkel–Holstein Hamiltonian is diagonalized in the specific points of Brillouin as relevant to spectroscopy. Absorption and emission spectra are then calculated assigning a Gaussian band shape (σ = 0.5 eV) to each transition, weighted by the corresponding squared transition dipole moment, as detailed in the ESI, Section S5. Color as RGB-tuple is finally calculated starting from the absorption spectra by an in-house code. These RGBs define the simulated corresponding colors except for an arbitrary extinction coefficient (or a color depth, equivalently).

### 2.9 Absorption and Fluorescence Measurements

UV–vis absorption spectra were recorded with a PerkinElmer Lambda650 spectrophotometer. Fluorescence measurements were performed on an FLS1000 Edinburgh Fluorometer. For each sample, a small quantity of the solid dispersed in nujol oil was ground in a mortar, and then a thin layer of the material was applied on a quartz plate. Absorption spectra were collected in transmission mode with the light beam perpendicular to the sample. A quartz plate was used as a reference. Emission spectra were acquired by exciting at 450 nm the same thin layers used for absorption. The sample was slightly 45° off with respect to the excitation beam to minimize the interference from reflected light. To remove artifacts due to scattering and stray light, appropriate long-pass filters were inserted in the emission path.

## 3 Results

We first investigated the properties of the QA monomer. TD-DFT results (Figure 3a­(i)) demonstrate that the lowest energy excited state of QA is well separated from the higher excited states and has a sizable transition dipole moment. In order to discriminate the role of different functional groups and identify the nature of low-lying excited states, the QA molecule was first partitioned into 6 fragments, as shown in Figure 3b­(i). The Ω matrix in the top panel of Figure 3c­(i) safely ascribes the lowest transition to a delocalized π → π∗ excitation. Higher energy excited states, with a localized n → π∗ nature involving the carbonyl units (see bottom of Figures 3c­(i) and S6), are optically dark, as expected. When embedding the monomer in the electrostatic field generated by the atomic charges of the surrounding medium, the lowest energy transition is stabilized by ∼0.2 eV, while its nature is not affected.

For a specific cluster obtained from the βQA structure along axis *b*, Figure 3 shows the evolution of the excited states from the monomer to a dimer and a hexamer. In the dimer, the same partitioning of the molecular units as performed in the monomer is adopted, while the hexamer is partitioned into molecular units. Accordingly, in the electron–hole correlation analysis, excited states with (delocalized intermolecular) excitonic nature would generate block-diagonal (dimer, Figure 3c­(ii)) or diagonal (hexamer, Figure 3c­(iii)) elements in the Ω matrix. Intermolecular CT states are signaled by nonvanishing off-diagonal elements or off-diagonal blocks.

In the dimer (Figure 3a­(ii)), in line with Kasha’s exciton model for H-aggregates, the two low-lying monomer excitations recombine into two states of different energy, with all oscillator strength collapsed into the highest energy state. At higher energy, two dark transitions appear with dominant CT character (see Figure 3c­(ii)). A similar behavior is observed for the hexamer, where, of course, each manifold contains 6 states. The same analysis is performed on other clusters: the cluster obtained as γQA along the *b* direction shows H-aggregate behavior; the βQA cluster along the a+b direction, and the γQA cluster along $\frac{1}{2}\left[a+b\right]$ and $\frac{1}{2}\left[a-b\right]$ ) directions all show J-aggregate behaviors (see Figures S12 and S14).

In general, both Frenkel and CT excitons can play a role in optical spectra for molecular crystals and aggregates.^39,40,43,92–95^ CT excitons typically have negligible oscillator strengths, but they can interact with nearby excited states, leading to important spectroscopic effects.^43^ In the case of acene crystals, for example, their role has been discussed in the literature and considered in computational perspectives.^38,96,97^ In line with previous work,^28,73^ our results confirm that CT states do not play any significant role in the low-energy optical properties of QA (Figure 3). Accordingly, we will not discuss CT states any further and will focus on the low-energy excited state manifold with a well-defined exciton nature.

Following the strategy described in the Methods section, from the analysis of TD-DFT results on embedded clusters, we obtain a reliable estimate of *J*-couplings between nearest-neighbor molecules. Relevant results for the two crystals are reported in Table 1. In the same table, we also show the other parameters of the Frenkel–Holstein model, obtained following the approaches detailed in Methods.

**Table 1:** Model Parameters^a^

phase	βQA	γQA
ϵ_0_	2.43	2.43
Δ	0.160	0.145
*J*_H_	–0.011	–0.008
*J*_π_	0.009	0.019
ω	0.168	0.168
*g*	0.120	0.120

a All of the values are in eV. *J*_π_ and *J*_H_ correspond to the two possible excitonic interactions; see text.

Vibrational degrees of freedom enter the model in terms of molecular vibrations modulating on-site energies in the classical Frenkel–Holstein model. Accounting for a single effective molecular vibration on each site with frequency ω_eff_, the Hamiltonian reads:

H^=∑i{[ϵ0+D−g(a^i†+a^i)]b^i†b^i+ℏωeff(a^i†a^i+12)+∑jJij(b^i†b^j+b^j†b^i)} 6

*a*_*i*_^†^ and *a*_*i*_ are the vibrational creation and annihilation operators for the effective vibrational mode on the *i*th molecule, *b*_*i*_^†^ and *b*_*i*_ are the creation and annihilation operators for the electronic excitation, *g* is the vibronic coupling constant, and *J*_*i* *j*_ measures the coupling between the molecules on the site *i* and *j*. As detailed above, we only account for excitonic interactions between nearest-neighbor sites linked by either hydrogen bonds or π stacking acting in the two-dimensional crystallographic planes of interest for the two crystals. The single-mode approximation is often adopted in the framework of the Frenkel-Holstein model.^43^ To further validate this approximation, Figure S3 in ESI shows the vibronic coupling strength partitioned into the different contributions. It is clear that the coupling strength clusters around the frequency of the effective mode. The 2D periodic Hamiltonian written in the reciprocal space is fully reported in the Methods, eqs 4 and 5.

To start with, we consider the electronic problem, setting *g* = 0. In these conditions, only the electronic wavevector is of relevance. For the βQA phase with a single molecule per unit cell, the first line in eq 4 (Methods) defines the energy of the exciton in the momentum space. Results are plotted in Figure 4. In the γQA phase, the presence of two molecules per unit cell leads to a slightly more complex problem, with the first two lines in eq 5 (Methods) defining a two-dimensional Hamiltonian at each point of the Brillouin zone. The diagonalization of the two-dimensional matrix leads to two exciton bands, as plotted in Figure 4. The ground state in the electronic model is a single point at **K** = **k** = 0, and the selection rule of optical spectroscopy, Δ**K** = 0, allows us to immediately recognize the states that can be reached upon photoexcitation, marked with a black star in the right-hand side panels of Figures 4. In the γQA phase, the two states reached upon photoexcitation have very similar energies and transition dipole moments. After photoexcitation, according to the Kasha rule,^98^ the system typically relaxes very quickly to the lowest energy state in the lowest exciton surface (marked as a white star in the right-hand panels of Figure 4a,b). In both polymorphs, the Kasha state is located at the border of the Brillouin zone, so emission is forbidden. However, this result is strictly valid only in the absence of vibrational coupling.

**Figure 4:**
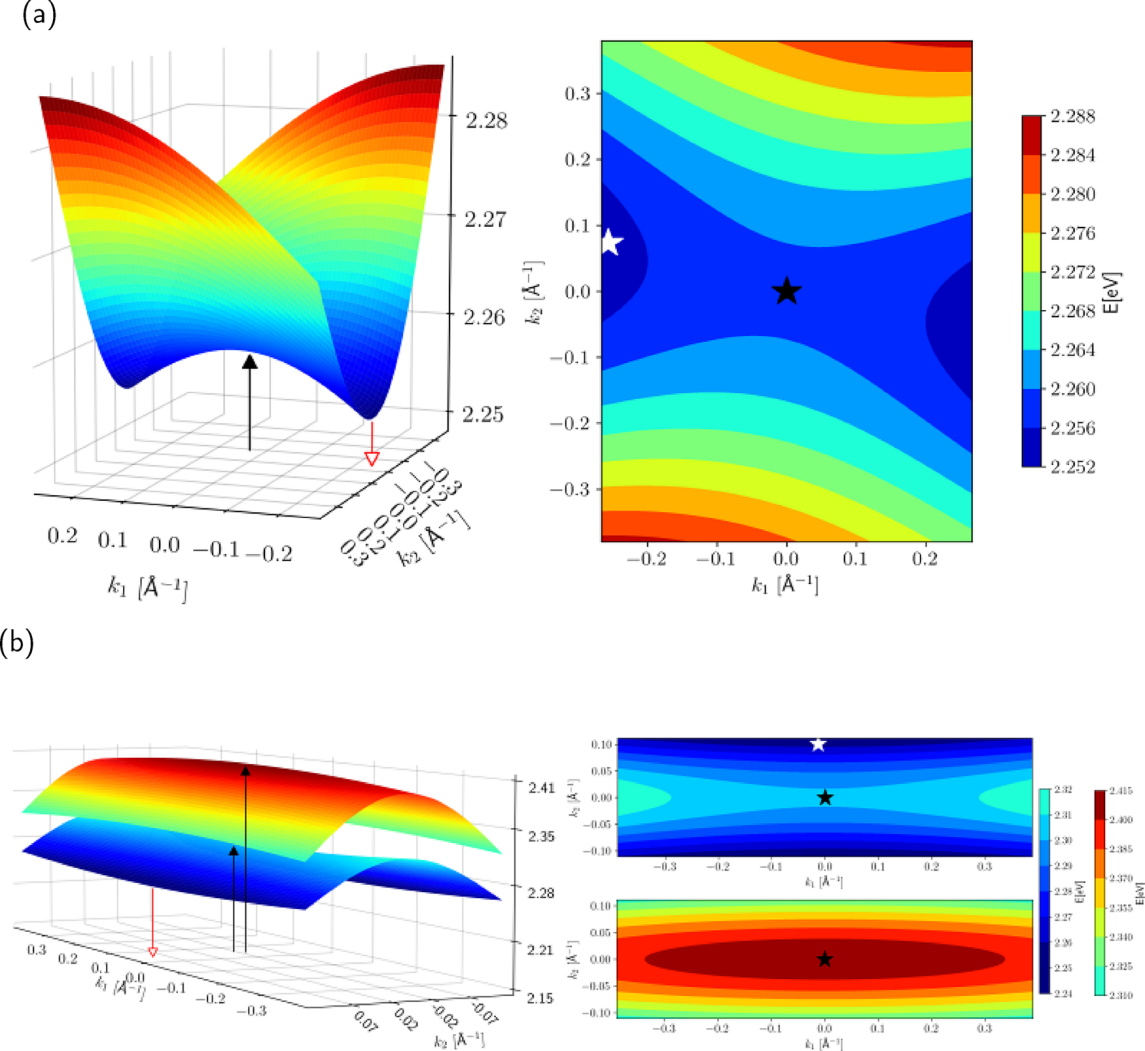
Electronic energy surfaces for the exciton states of βQA (a) and γQA (b). On the left side: 3D representation, and on the right: 2D colormap representation. The colorscale is displayed on the right. The black arrow (left) and the black stars (right) represent the center zone, the sole point at which the absorption takes place. The red arrow (left) and white star (right) represent the points of the (forbidden) emission.

When vibrational coupling is switched on, the wavevector is the sum of the electronic and vibrational wavevectors, **K = k + q**. In the low temperature limit, vibrational states are not thermally populated, and the ground state is located at **K** = **k** = 0. States reached upon absorption can have population on the vibrational levels, but the optical selection rules impose that **K** = **k** + **q** = 0 or **k** = −**q** = – ∑_ν_ *n*_ν_ **q**_ν_, where *n*_ν_ are the occupation numbers for the vibrational wavevectors ***q***_ν_. Emission occurs from the Kasha state, the lowest energy state in the excited state manifold, characterized by a total wavevector **K**_Kasha_. Upon emission, the optical selection rule imposes that a state is reached in the ground state manifold with the same total wavevector, **K**_Kasha_. Accordingly, since the electronic ground state has **k** = 0, the relevant state in the ground state manifold has ∑_ν_ *n*_ν_ **q**_ν_ = **K**_Kasha_. To calculate spectra, we considered 2D aggregates comprising 4 × 4 unit cells (16 and 32 molecules for βQA and γQA, respectively), limiting the total number of vibrational quanta to 3 (we explicitly checked the quasi-convergence of calculated spectra). Temperature effects were tested, accounting for a Boltzmann energy distribution, but only marginal broadening effects were detected.

Focusing on absorption, vibronic coupling gives rise to a manifold of vibronic states on each exciton surface and is therefore responsible for the appearance of a vibronic structure. The role of molecular vibrations is even more important in emission. Specifically, the ground state is itself dressed by vibrational modes so that states are present in the ground state manifold at the border of the Brillouin zone, making emission possible. Emission originates from the lowest energy states in the excited state manifold, and ends up in states with finite vibrational population on the ground state manifold. The emission band edge is therefore red-shifted with respect to the absorption.

Figure 5 compares experimental and simulated spectra. For both polymorphs, absorption and emission band shapes agree very well with experiment. The agreement is particularly striking since there are no adjustable parameters in the adopted model. In fact, all model parameters are extracted from ab initio simulations, with the only exception of ϵ_0_, the exciton energy, which is extracted from experimental data relevant to QA in solution. This choice is motivated by the well-known problem of TD-DFT in the calculation of accurate absolute transition energies, as already discussed for QA.^99^ Relying on TD-DFT results, the transition energies would be overestimated by roughly 0.9 eV (see ESI Figure S4 and Tables S2–S17).

**Figure 5:**
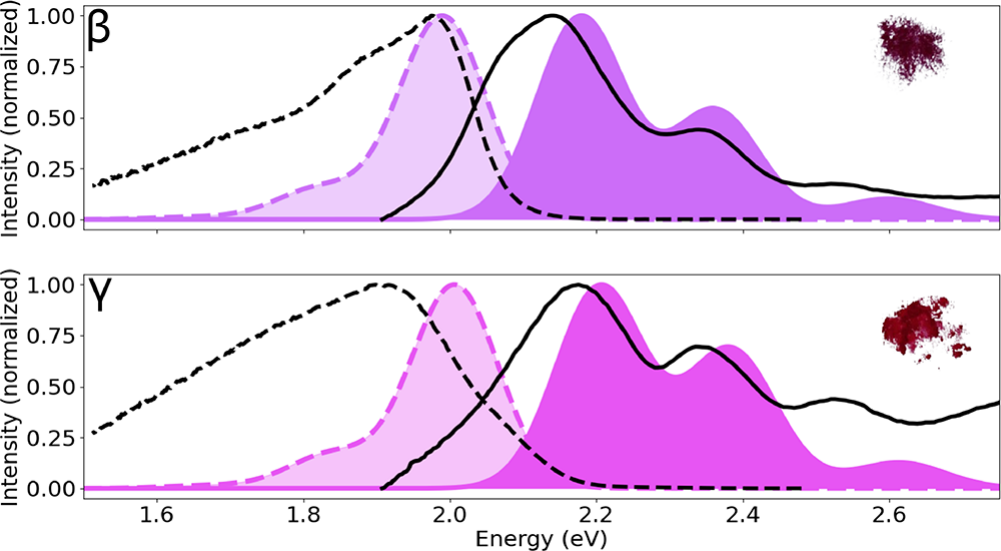
Calculated absorption and emission spectra of βQA (top) and γQA (bottom) of QA compared to experimental data (this work). Experimental spectra are shown as black thick lines, while calculated spectra are filled in dark purple (βQA, RGB: 204,109,249) and magenta­(γQA, RGB: 23,185,243), i.e., the colors calculated using the corresponding simulated absorption spectra. Areas under the emission spectra are filled with a variant of the corresponding absorption colors with arbitrary transparency. Solid lines are used for absorption spectra and dashed lines for emission spectra. In the insets on the right-top side, we show photographs of the crystal powders taken for the two phases.

To reproduce experimental spectra, a fairly large width is assigned to the Gaussian bands associated with each transition. This effectively accounts for inhomogeneous broadening phenomena that are not included in the FE model. Specifically, low-frequency vibrational modes are responsible for broadening, as well as disorder and structural defects that are absent in the adopted perfect crystalline model.

The major discrepancy between calculated and experimental spectra is seen in emission, with the experimental band showing a broad component in the red portion of the spectrum that is missing in the simulation. This is ascribed to the presence of defects and trap states in the sample that may contribute to the actual emission and are not accounted for in our model for a perfect crystal. In any case, the overall quality of the simulated spectra is very satisfactory and validates the proposed approach.

Looking at Figure 5, we notice the prominent role of vibrational states in determining the optical signatures of QA in absorption and emission. This suggests that quantum treatment of coupled excitonic-vibrational states, as suggested in our hybrid approach, is a fundamental step toward reliable spectra simulation. Conversely, electronic methods based on QM or QM/MM methods would fail to capture these features even though they offer an accurate description of the embedding.

Based on the simulated absorption spectra, it is possible to simulate the corresponding color. These colors are reported in Figure 5 as a filled area under the absorption spectra. Unfortunately, reobtaining quantitative components in color space from experimental spectra is not straightforward mainly due to the scattering artifacts appearing in the high-energy side of the bands. In addition, the effective color appearance perceived by human eyes for the two polymorphs is not necessarily easy to quantify, nor can it be accurately reproduced on the reading format (paper or screen). However, we are able to capture the amount of blue vs red hue dominance in βQA vs γQA, respectively.

## 4 Discussion and Conclusions

The evolution of the optical properties of a molecule from solution to a crystalline phase is highly nontrivial, being dominated by intermolecular interactions with the appearance of collective excitations or, in other terms, of delocalized excited states. Molecular vibrations add a layer of complexity in this scenario and, as discussed above, profoundly alter the spectral properties in terms of band shapes and/or emissive properties. Here, we introduced a comparatively simple and computationally accessible hybrid modeling protocol, relying on the parametrization of the Frenkel–Holstein Hamiltonian against quantum chemical calculations. Specifically, periodic DFT was adopted to optimize the crystallographic geometry and extract effective atomic charges. TD-DFT results on the isolated molecule are exploited to parametrize the Holstein coupling. TD-DFT calculations on the embedded molecule and clusters of increasing size are finally exploited to extract the exciton model parameters. The only phenomenological inputs are the refractive index of the crystal and ϵ_0_, the absorption frequency of the molecule in solution that enters to recalibrate the TD-DFT absolute energies. Specifically, the squared refractive index enters into the renormalization of the exciton couplings as extracted from TD-DFT, in line with recent theoretical discussions.^100^ Indeed, simulated spectra calculated without accounting for dielectric screening poorly agree with experiment (see ESI Figure S19). For ϵ_0_, we prefer to refer to the experimental transition energy of QA in solution, rather than introduce an arbitrarily rigid shift of the TD-DFT energies.

Exciton couplings are often estimated in the point-dipole approximation. In this approximation, the estimated *J* (in Table S20) are much larger than in our current approach, leading to a poor agreement between calculated and experimental spectra (Figure S20).

The approach is validated against the interesting case of QA, a molecular system featuring two polymorphs with distinctively different optical spectra (and colors). In the process, we collected a set of new experimental absorption and emission spectra for the β and γ phases of QA, in order to fill a gap in the available literature data for these crystals. The agreement between the simulated and experimental spectra is very good, demonstrating the validity of the method. The proposed multiscale approach applies to molecular crystals, where CT transitions are marginally involved in low-energy excited states. In principle, it could be automated once a dictionary of symmetries is established. Finally, having access to experimental absorption spectra in solution may help to fine-tune ϵ_0_.

Recently, Giannini et al. have addressed the spectral properties of a nonfullerene acceptor labeled Y6.^41^ Their model includes FEs, CTs, and vibrational states, and it is based on a similar hybrid strategy of a model Frenkel–Holstein Hamiltonian supplemented by ab initio calculations. They were able to describe the spectral evolution from the solution to the thin film and the crystal. For Y6, it was shown that CTs and FEs can strongly mix due to a favorable energy alignment. By accounting for their combined mixing with vibronic states, the authors accurately reproduced the absorption in the visible and infrared. Although emission spectra were not addressed, their strategy proved to be successful in explaining the photophysics of Y6 and interpreting the additional features observed in the experiments.

Multiscale flavors were also suggested in a conceptual workflow proposed by Bondarenko et al.,^101^ with the aim to simulate optical properties of a large supramolecular aggregate of dyes. Their iterative multiscale approach combines molecular dynamics and quantum mechanical exciton modeling. However, vibrational states were not included in their model, which was limited to the case of J-aggregates with large excitonic coupling. A multiscale bottom-up scheme has also been suggested to compute the nonlinear properties of a molecular crystal.^102^

Our work is placed in this promising direction of multiscale approaches. An important novelty is the actual simulation of the emission spectra. In the case of QA, we observe that emission is permitted only as a consequence of mixing with vibronic states. Our robust parametrization, combined with the proper building of a model Hamiltonian, well explains the different absorption and emissions measured for the polymorphs of QA, hence paving the way to a rationalization of crystallochromism of molecular condensed phases.

## Supplementary Material



## Notes

Crystal structure analysis of γ and β phases of QA; DFT and TDDFT calculations on both isolated and embedded monomer, dimers and hexamers; details on the fitting procedure used to obtain the parameters of the effective models; mathematical derivation of the Hamiltonians; experimental spectra and additional simulated spectra (PDF)
