# Supplementary material for: Crystallochromism: A Hybrid Model for the Spectral Properties of Quinacridone Polymorphs
Source: J Chem Theory Comput. 2025 Oct 29;21(21):11179–89. doi: 10.1021/acs.jctc.5c01022 (PMC12613315; doi:10.1021/acs.jctc.5c01022)
Supplement: Supplementary file 1 [file ct5c01022_si_001.pdf]

ELECTRONIC SUPPORTING INFORMATION

Crystallochromism: a hybrid model for the spectral properties of Quinacridone polymorphs

Lorenzo Savi [1], Matteo Masino[1], Anna Painelli*[1], and Luca Grisanti*[2,3]

[1]Department of Chemistry, Life Science and Environmental Sustainability, Parma University, Parco Area delle Scienze 17/A, 43124 Parma, Italy

[2]Division of Theoretical Physics, Ruđer Bošković Institute, Bijenička cesta 54, 10000 Zagreb, Croatia

[3]CNR - Istituto Officina dei Materiali (IOM) c/o SISSA (International School for Advanced Studies), via Bonomea 256, 34136 Trieste, Italy

Note: citation numbers refer to the specific bibliography of this document, which anyway is included in the main paper.

Contents

1 Crystal structures **4**

2 TD-DFT excitations and analysis **5**

2.1 Monomer 5

2.2 Dimers 11

2.3 Hexamers 19

3 Linear aggregate fitting **31**

4 Model Hamiltonians **32**

4.1 βQA 32

4.2 γQA 32

5 Simulation of absorption and emission spectra **36**

6 Emission and absorption spectra **36**

List of Figures

S1 βQA crystal structure . . . 4
S2 γQA crystal structure . . . 4
S3 Vibronic couplings g at normal mode frequencies (monomer in vacuum). . . . 6
S4 Exciton energy energy adjusment . . . 7
S5 Convergence with the QM/MM embedding . . . 8
S6 βQA Ω matrices excitation analysis: monomer (embedded) . . . 9
S7 γQA Ω matrices excitation analysis: monomer (embedded) . . . 10
S8 βQA H-Bond Ω matrices excitation analysis: dimer (embedded) . . . 12
S9 βQA π-stacked Ω matrices excitation analysis: dimer (embedded) . . . 14
S10 γQA H-Bond Ω matrices excitation analysis: dimer (embedded) . . . 16
S11 γQA π-stacked Ω matrices excitation analysis: dimer (embedded) . . . 18
S12 γQA H-Bond Ω matrices excitation analysis: hexamer (embedded) . . . 21
S13 βQA π-stacked Ω matrices excitation analysis: hexamer (embedded) . . . 24
S14 γQA H-Bond Ω matrices excitation analysis: hexamer (embedded) . . . 27
S15 βQA π-stacked Ω matrices excitation analysis: hexamer (embedded) . . . 30
S16 Monomer to hexamer transitions in the embedded βQA π-stacked aggregates . . . 31
S17 Fitting of linear aggregates with model Hamiltonian. . . . 32
S18 Experimental spectra . . . 36
S19 QA spectra using Js without refractive index correction . . . 37
S20 QA spectra using Js with the point dipole approximation . . . 38

List of Tables

S1 DFT Gas phase frequency calculation . . . 5
S2 Monomer βQA excitations . . . 8
S3 Monomer γQA excitations . . . 9
S4 βQA H-Bond monodimensional aggregate: dimer (vacuum) . . . 11
S5 βQA H-Bond monodimensional aggregate: dimer (embedded) . . . 11
S6 βQA π-stacked monodimensional aggregate: dimer (vacuum) . . . 13
S7 βQA π-stacked monodimensional aggregate: dimer (embedded) . . . 13
S8 γQA H-Bond monodimensional aggregate: dimer (vacuum) . . . 15
S9 γQA H-Bond monodimensional aggregate: dimer (embedded) . . . 15
S10 γQA π-stacked monodimensional aggregate: dimer (vacuum) . . . 17
S11 γQA π-stacked monodimensional aggregate: dimer (embedded) . . . 17
S12 βQA H-Bond monodimensional aggregate: hexamer (vacuum) . . . 19
S13 βQA H-Bond monodimensional aggregate: hexamer (embedded) . . . 20
S14 βQA π-stacked monodimensional aggregate: hexamer (vacuum) . . . 22
S15 βQA π-stacked monodimensional aggregate: hexamer (embedded) . . . 23
S16 γQA H-Bond monodimensional aggregate: hexamer (vacuum) . . . 25
S17 γQA H-Bond monodimensional aggregate: hexamer (embedded) . . . 26
S18 γQA π-stacked monodimensional aggregate: hexamer (vacuum) . . . 28
S19 γQA π-stacked monodimensional aggregate: hexamer (embedded) . . . 29

S20 Comparison between possible choices for the excitonic couplings 37

1 Crystal structures

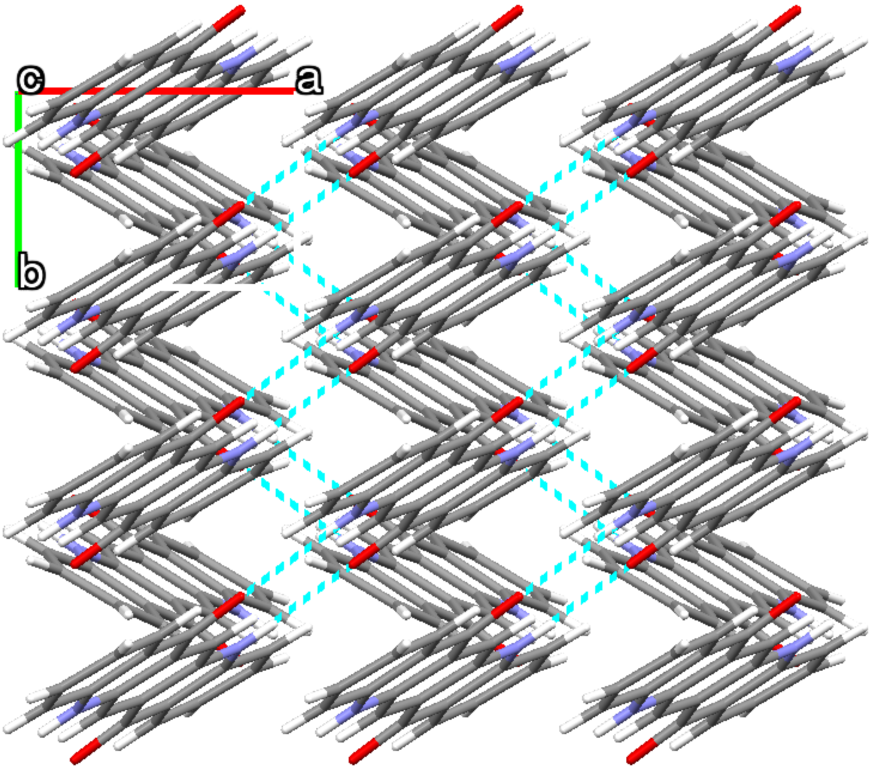

Figure S1: β**QA crystal structure.** In βQA [1] each molecule is bonded to the two nearest neighbor molecules via two hydrogen bonds each, forming alternate chains that are not parallel. Half of the chains run in the $[110]$ direction and the other half run in the $[1\bar{1}0]$ direction. All chains are equivalent by symmetry. The molecules form stacks through $\pi\cdots\pi$ interactions in the b direction, in 2D layers parallel to the 001 direction that are held together by Van der Waals interactions. To sum up, the three-dimensional crystal can be described as set of non-interacting 2D sheets, held together by independent hydrogen bonds and π stacking interactions.

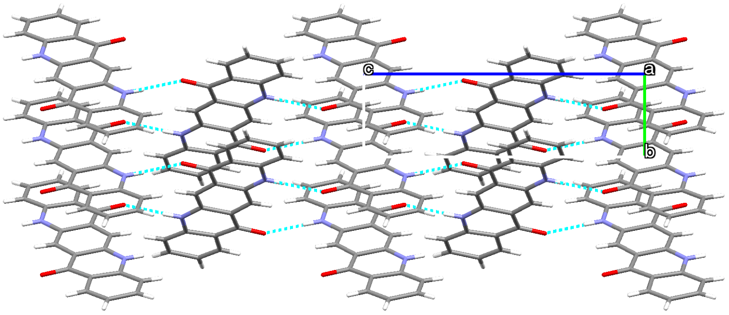

Figure S2: γ**QA crystal structure.** In γQA [1], each molecule is connected through a single hydrogen bond to the four nearest neighbor molecules, forming a criss-cross pattern. The hydrogen-bond network develops along a unique direction roughly parallel to the $[001]$ direction. Alongside the hydrogen bonds network, the molecules form stacks through $\pi\cdots\pi$ interactions in the b direction. The crystal can be described as non-interacting 2D sheets parallel to the $[100]$ direction, held together by hydrogen bonds and π stacking, but at variance with βQA , the interactions are not independent and each molecule that belongs to a sheet is part of the unique hydrogen-bond network of that sheet.

.

2 TD-DFT excitations and analysis

TD-DFT calculations were performed with `Orca 5` at ωB97X-D3BJ (equivalently ωB97X for TDDFT transitions) with def2-TZVP basis set. See Methods for all details. For bot βQA and γQA we present additional figures and tables for:

- monomers in-vacuum (include frequency and vibronic coupling calculations) and embedded (including evaluation of embedding)
- dimers (H-Bond and π-stacked), both in-vacuum (tables only) and embedded
- hexamers (H-Bond and π-stacked), both in-vacuum (tables only) and embedded

2.1 Monomer

Table S1: **DFT Gas phase frequency calculation.** Vibrational modes, Huang-Rhys factors, frequency in eV, coupling relative to the vibronic spectrum for the first transition of QA. Harmonic mode calculated on the optimized ωB97X-D3BJ/def2-TZVP structure in vacuum with the ωB97X-D3BJ/def2-TZVP level of theory; vibronic progression calculated with the AHAS (Adiabatic Hessian After a Step) method and the ωB97X-D3BJ/def2-tzvp level of theory.

n	S	ω(eV)	g(eV)	n	S	ω(eV)	g(eV)	n	S	ω(eV)	g(eV)
**1**	0.000	0.004	0.000	**35**	0.000	0.092	0.000	**69**	0.035	0.165	0.031
**2**	0.000	0.007	0.000	**36**	0.000	0.093	0.000	**70**	0.000	0.166	0.000
**3**	0.000	0.008	0.000	**37**	0.002	0.095	0.004	**71**	0.000	0.168	0.000
**4**	0.000	0.014	0.000	**38**	0.000	0.097	0.000	**72**	0.175	0.169	0.071
**5**	0.000	0.015	0.000	**39**	0.000	0.097	0.000	**73**	0.000	0.172	0.001
**6**	0.000	0.017	0.000	**40**	0.000	0.103	0.000	**74**	0.000	0.172	0.000
**7**	0.000	0.024	0.000	**41**	0.000	0.103	0.000	**75**	0.000	0.177	0.000
**8**	0.000	0.024	0.000	**42**	0.000	0.104	0.000	**76**	0.054	0.183	0.043
**9**	0.009	0.029	0.003	**43**	0.000	0.110	0.000	**77**	0.000	0.187	0.000
**10**	0.045	0.030	0.006	**44**	0.000	0.110	0.000	**78**	0.000	0.189	0.000
**11**	0.000	0.034	0.000	**45**	0.004	0.111	0.007	**79**	0.006	0.190	0.014
**12**	0.000	0.035	0.000	**46**	0.000	0.113	0.000	**80**	0.000	0.193	0.000
**13**	0.000	0.038	0.000	**47**	0.006	0.116	0.009	**81**	0.028	0.194	0.033
**14**	0.014	0.043	0.005	**48**	0.008	0.116	0.010	**82**	0.000	0.199	0.000
**15**	0.000	0.049	0.000	**49**	0.000	0.117	0.002	**83**	0.001	0.205	0.007
**16**	0.000	0.052	0.000	**50**	0.000	0.123	0.000	**84**	0.000	0.207	0.001
**17**	0.000	0.054	0.000	**51**	0.000	0.125	0.000	**85**	0.004	0.207	0.013
**18**	0.000	0.055	0.000	**52**	0.000	0.125	0.000	**86**	0.000	0.211	0.000
**19**	0.000	0.056	0.000	**53**	0.000	0.126	0.000	**87**	0.002	0.211	0.008
**20**	0.000	0.056	0.000	**54**	0.000	0.126	0.000	**88**	0.000	0.214	0.003
**21**	0.003	0.058	0.003	**55**	0.003	0.132	0.007	**89**	0.000	0.218	0.000
**22**	0.001	0.058	0.002	**56**	0.000	0.133	0.000	**90**	0.034	0.220	0.040
**23**	0.000	0.060	0.000	**57**	0.001	0.140	0.005	**91**	0.000	0.396	0.002
**24**	0.014	0.068	0.008	**58**	0.000	0.141	0.000	**92**	0.000	0.396	0.002
**25**	0.000	0.069	0.000	**59**	0.000	0.143	0.000	**93**	0.000	0.398	0.001
**26**	0.000	0.069	0.000	**60**	0.013	0.144	0.017	**94**	0.000	0.398	0.003
**27**	0.000	0.070	0.000	**61**	0.000	0.147	0.000	**95**	0.000	0.398	0.001
**28**	0.018	0.074	0.010	**62**	0.040	0.147	0.029	**96**	0.000	0.398	0.006
**29**	0.000	0.078	0.000	**63**	0.000	0.152	0.000	**97**	0.000	0.399	0.000
**30**	0.000	0.079	0.000	**64**	0.030	0.154	0.027	**98**	0.000	0.399	0.000
**31**	0.001	0.081	0.002	**65**	0.009	0.157	0.015	**99**	0.000	0.401	0.000
**32**	0.000	0.087	0.000	**66**	0.000	0.158	0.000	**100**	0.000	0.401	0.002
**33**	0.000	0.087	0.000	**67**	0.000	0.161	0.000	**101**	0.000	0.456	0.001
**34**	0.009	0.089	0.008	**68**	0.013	0.161	0.018	**102**	0.002	0.456	0.018

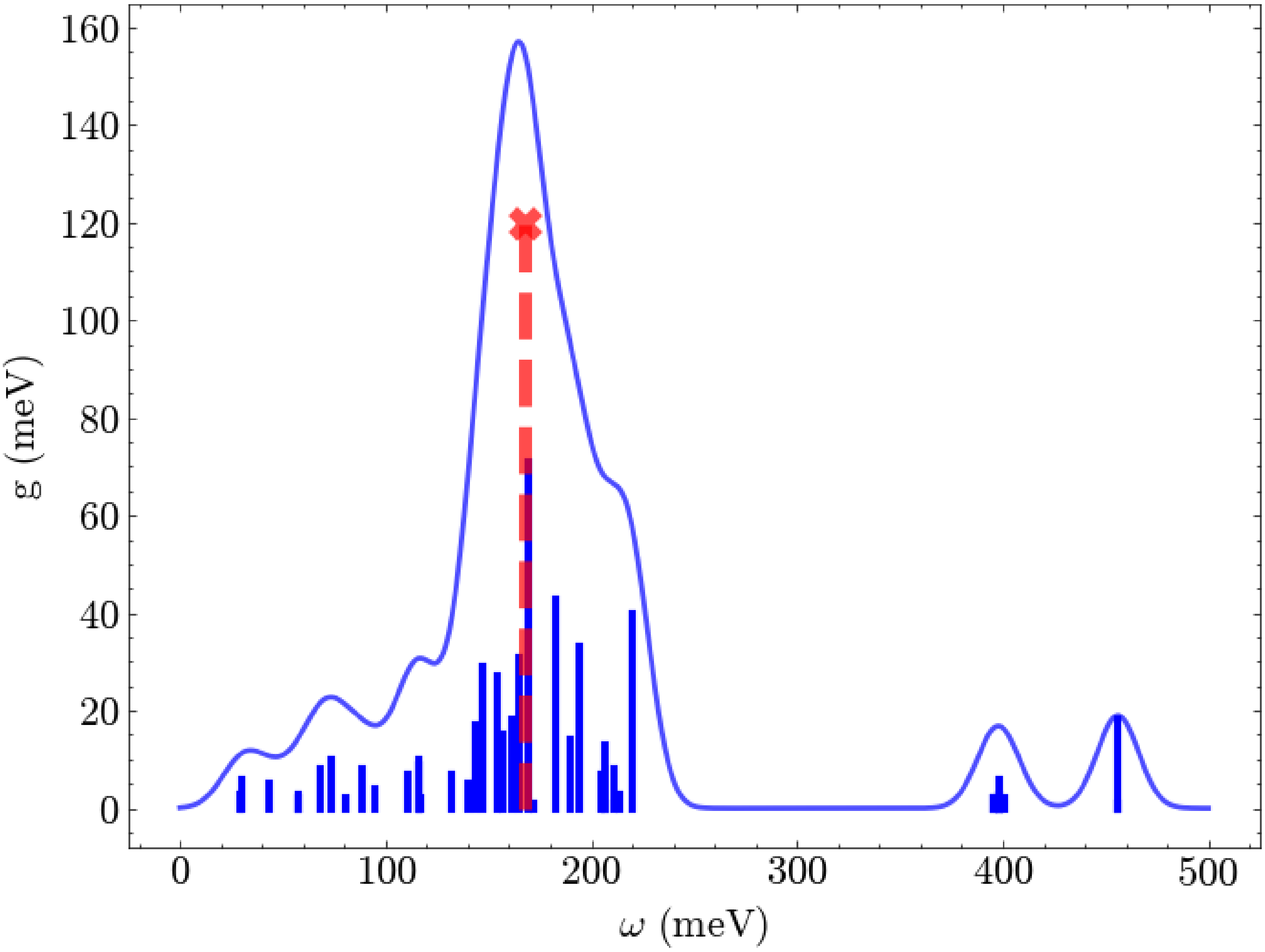

Figure S3: **Vibronic couplings g at normal mode frequencies (monomer in vacuum)..** g_i at each corresponding ω_i in meV for the vibrational modes analyzed in Tab.S1. Bars: g values as shown in the table. Blue line: convolution of the bars with a Gaussian lineshape with $\sigma = 0.01eV$ to simulate a frequency distribution. Red line and red cross: coupling mode at the average effective mode frequency ω.

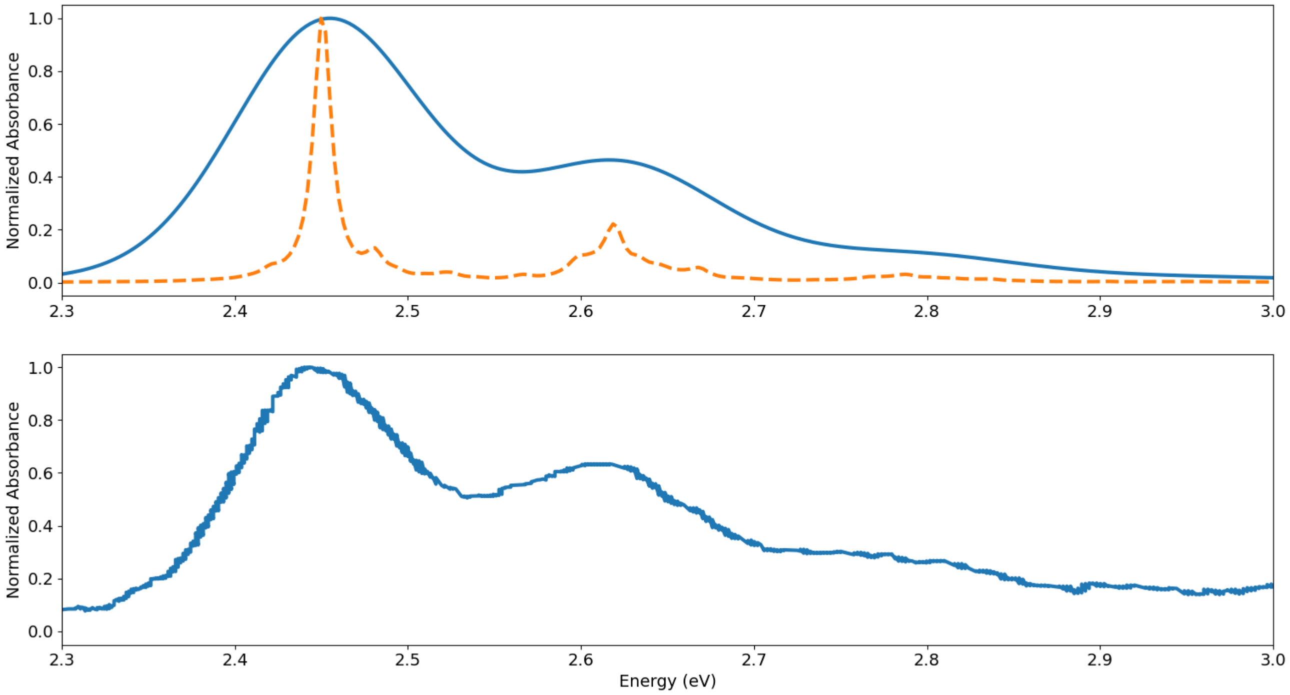

Figure S4: **Exciton energy energy adjusment.** Top: spectra simulated with `AHAS` (Adiabatic Hessian After a Step) technique. Harmonic mode calculated on the optimized structure at the ωB97X-D3BJ/def2-TZVP level of theory as in Table S1. Yellow: the raw spectrum obtained from Orca. Blue: the same spectra broadened by convolution with a sum of Gaussian function with $\sigma = 0.05$eV. Both spectra were redshifted by 0.92 eV to maximize the overlap with the experimental spectrum, correspondimng to an ϵ_0 value of 2.43 eV. Bottom: experimental spectrum of QA in Dioxane [2].

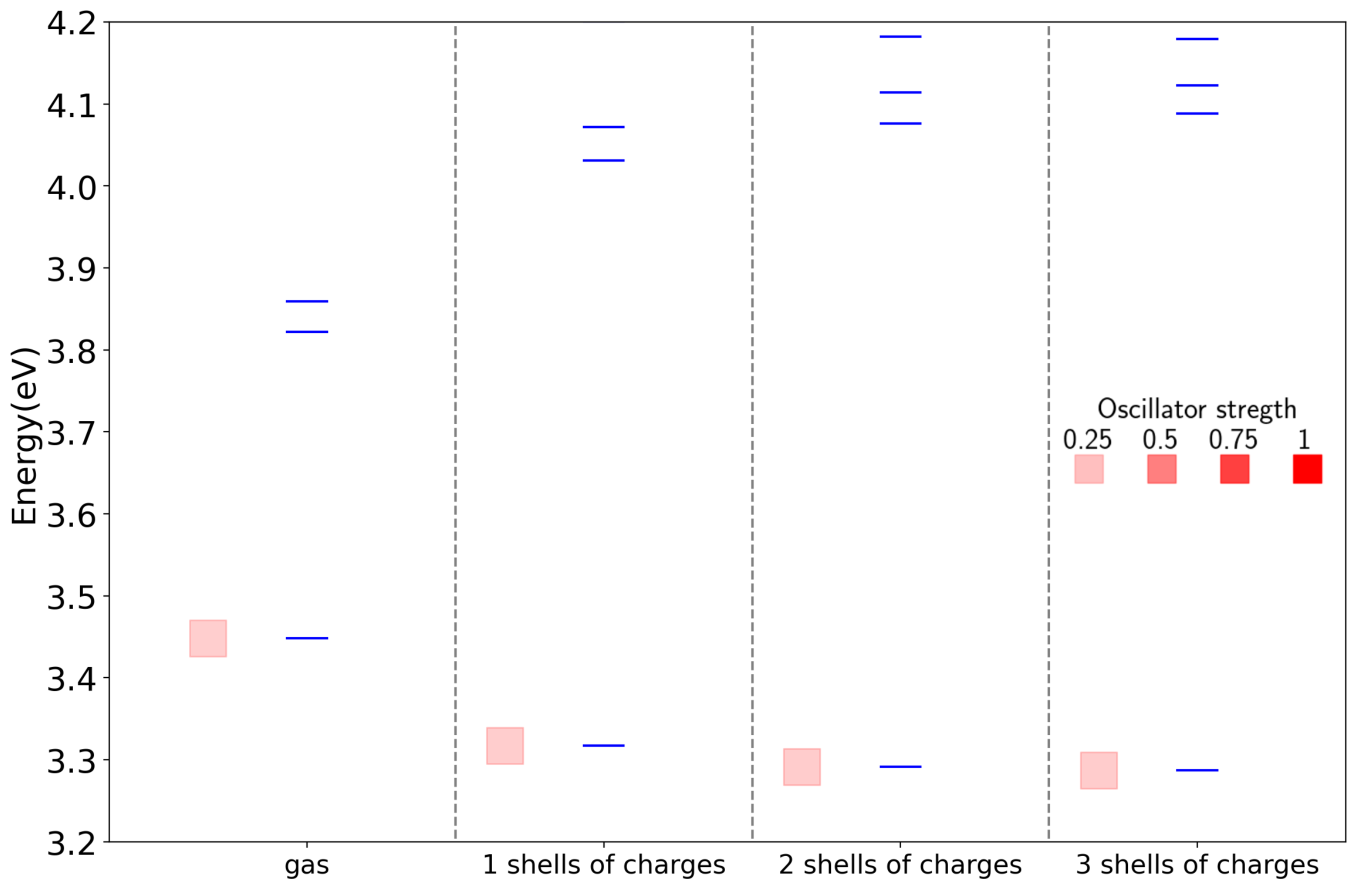

Figure S5: **Convergence with the QM/MM embedding.** βQA monomer in gas phase, one, two and three shells of molecular charges (see Methods). In blue the transition energy; in red the oscillator strength. ωB97X functional, def2-TZVP basis set.

**a)** State	Energy (eV)	Wavelength (nm)	Oscillator Strength	μ_x(au)	μ_y(au)	μ_z(au)
1	3.44791	359.6	0.194233	-1.251360	0.785020	0.342590
2	3.82177	324.4	0.000000	-0.000070	0.000040	0.000030
3	3.85967	321.3	0.000313	-0.029600	0.029150	0.039840
4	4.29990	288.4	0.000000	0.000000	0.000000	0.000020
5	4.40580	281.4	0.085870	0.251170	-0.214530	-0.828540
6	5.22019	237.5	0.000000	-0.000010	0.000090	0.000560
**b)** State	Energy (eV)	Wavelength (nm)	Oscillator Strength	μ_x(au)	μ_y(au)	μ_z(au)
1	3.28738	377.2	0.199489	-1.292640	0.810850	0.386070
2	4.08836	303.3	0.000001	-0.002320	0.002410	0.001910
3	4.12371	300.7	0.000146	-0.021360	0.020670	0.023750
4	4.17931	296.7	0.000007	0.004410	-0.003160	-0.006430
5	4.30406	288.1	0.071344	0.250540	-0.203780	-0.756610
6	5.07989	244.1	0.001754	0.000460	0.011330	0.118150

Table S2: **Monomer βQA excitations.** Results for the βQA in-vacuum (a) and embedded (b) monomer obtained with TDDFT at ωB97X with def2-TZVP basis set. The calculation for the embedded monomer employ 3 layers of point-charges that simulate the βQA environment around the molecule. Rows marked in green correspond to the selected electronic states possessing excitonic features.

a) State	Energy (eV)	Wavelength (nm)	Oscillator Strength	μ_x(au)	μ_y(au)	μ_z(au)
1	3.43745	360.7	0.194425	0.537130	-0.362980	-1.374240
2	3.80076	326.2	0.000000	0.000290	-0.000170	-0.000290
3	3.83411	323.4	0.000154	-0.030600	0.025860	0.006180
4	4.29182	288.9	0.000000	-0.000630	-0.000070	0.000130
5	4.39957	281.8	0.084573	-0.775020	0.404400	0.143110
6	5.20749	238.1	0.000005	-0.004790	0.002210	-0.002930

b) State	Energy (eV)	Wavelength (nm)	Oscillator Strength	μ_x(au)	μ_y(au)	μ_z(au)
1	3.29241	376.8	0.202047	-0.563280	0.378990	1.433510
2	4.12187	300.8	0.000000	0.000210	-0.000180	0.000320
3	4.15014	298.8	0.000075	0.020920	-0.016080	0.006440
4	4.16512	297.7	0.000000	0.000390	-0.000500	-0.000270
5	4.28967	289	0.048471	-0.572430	0.301510	0.206540
6	5.06301	244.9	0.000003	-0.000020	-0.000260	-0.005110

Table S3: **Monomer γQA excitations.** Results for the γQA in-vacuum (a) and embedded (b) monomer obtained with TDDFT at ωB97X with def2-TZVP basis set. The calculation for the embedded monomer employ 3 layers of point-charges that simulate the γQA environment around the molecule. Rows marked in green correspond to the selected electronic states possessing excitonic features.

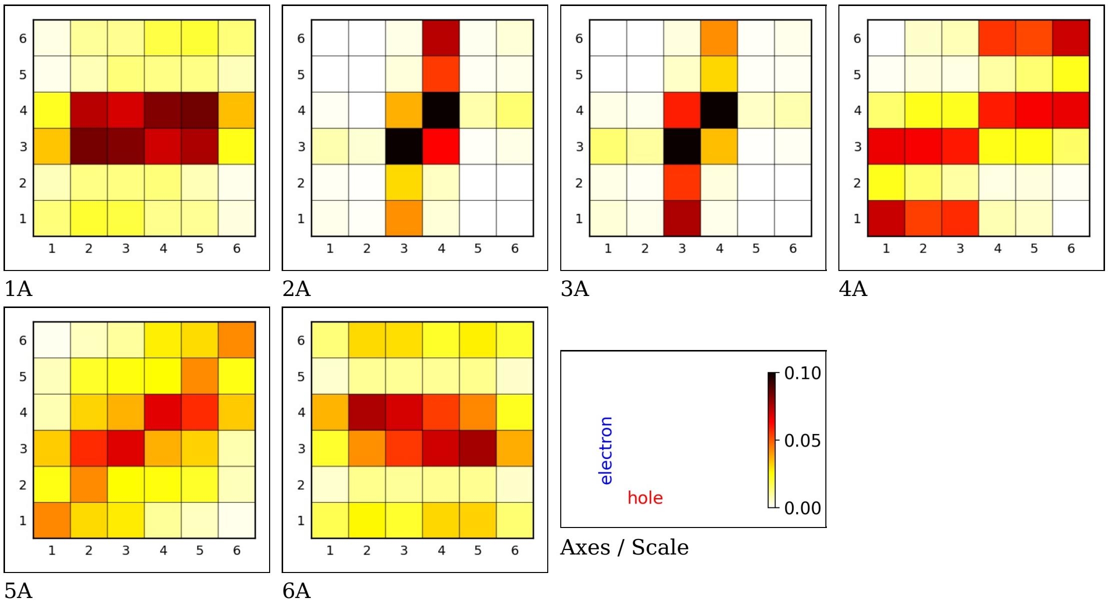

Figure S6: **βQA Ω matrices excitation analysis: monomer (embedded).** βQA embedded monomer electron-hole correlation analysis represented as Ω matrices as obtained from `TheoDORE` at ωB97X with def2-TZVP basis set. Excited states (1A, 2A, etc.) ordered from top left by rows. The colorscale is reported in the last box. [3].

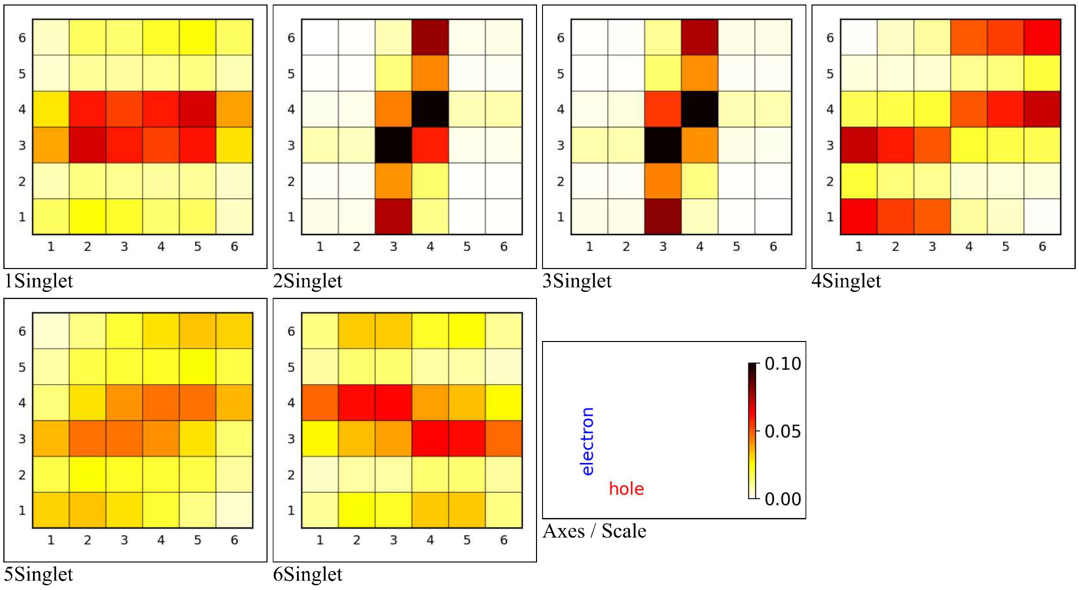

Figure S7: γ**QA Ω matrices excitation analysis: monomer (embedded).** γQA embedded monomer electron-hole correlation analysis represented as Ω matrices as obtained from `TheoDORE` at ωB97X with def2-TZVP basis set. Excited states (1A, 2A, etc.) ordered from top left by rows. The colorscale is reported in the last box. [3].

2.2 Dimers

State	Energy (eV)	Wavelength (nm)	Oscillator Strength	μ_x(au)	μ_y(au)	μ_z(au)
1	3.25838	380.5	0.475739246	2.02869	-1.27746	-0.46045
2	3.32478	372.9	0.000000031	-0.00052	0.00032	0.00011
3	3.81206	325.2	0.000198540	-0.01978	0.02533	0.03306
4	3.81209	325.2	0.000000971	-0.00135	0.00177	0.00233
5	4.16212	297.9	0.000000000	-0.00001	0.00001	0.00003
6	4.22884	293.2	0.000149094	-0.02498	0.01993	0.02044
7	4.22911	293.2	0.000000006	0.00016	-0.00019	0.00000
8	4.24318	292.2	0.011718787	-0.18620	0.08474	-0.26623
9	4.3323	286.2	0.000001039	-0.00123	0.00094	0.00272
10	4.33543	286.0	0.099420779	0.39206	-0.29582	-0.83355
11	4.57182	271.2	0.004930381	-0.16728	0.10935	0.06386
12	4.57281	271.1	0.000000223	0.00111	-0.00075	-0.00044

Table S4: β**QA H-Bond monodimensional aggregate: dimer (vacuum).** Results for the βQA gas-phase dimer obtained with TDDFT at ωB97X with def2-TZVP basis set. Rows marked in green correspond to the selected electronic states possessing excitonic features.

State	Energy (eV)	Wavelength (nm)	Oscillator Strength	μ_x(au)	μ_y(au)	μ_z(au)
1	3.125	396.8	0.471923692	2.06597	-1.29728	-0.46140
2	3.2014	387.3	0.001620569	0.11992	-0.07513	-0.02521
3	4.06316	305.1	0.000055956	-0.00314	0.00304	0.02330
4	4.14344	299.2	0.045359596	0.00972	-0.02581	-0.66789
5	4.15483	298.4	0.000615543	0.01764	-0.00942	0.07515
6	4.16045	298.0	0.000309917	0.01948	-0.01027	0.05055
7	4.23139	293.0	0.003202061	0.05909	-0.05110	-0.15743
8	4.23809	292.5	0.006853556	0.09168	-0.07512	-0.22795
9	4.23934	292.5	0.025663848	-0.18434	0.13973	0.43999
10	4.24379	292.2	0.045006957	-0.25053	0.18808	0.57857
11	4.60888	269.0	0.000191800	0.03235	-0.02081	-0.01481
12	4.62418	268.1	0.000148597	0.02717	-0.01781	-0.01601

Table S5: β**QA H-Bond monodimensional aggregate: dimer (embedded).** Results for the βQA embedded dimer obtained with TDDFT at ωB97X with def2-tzvp basis set and employing 3 layers of point-charges around the molecule. Rows marked in green correspond to the selected electronic states possessing excitonic features.

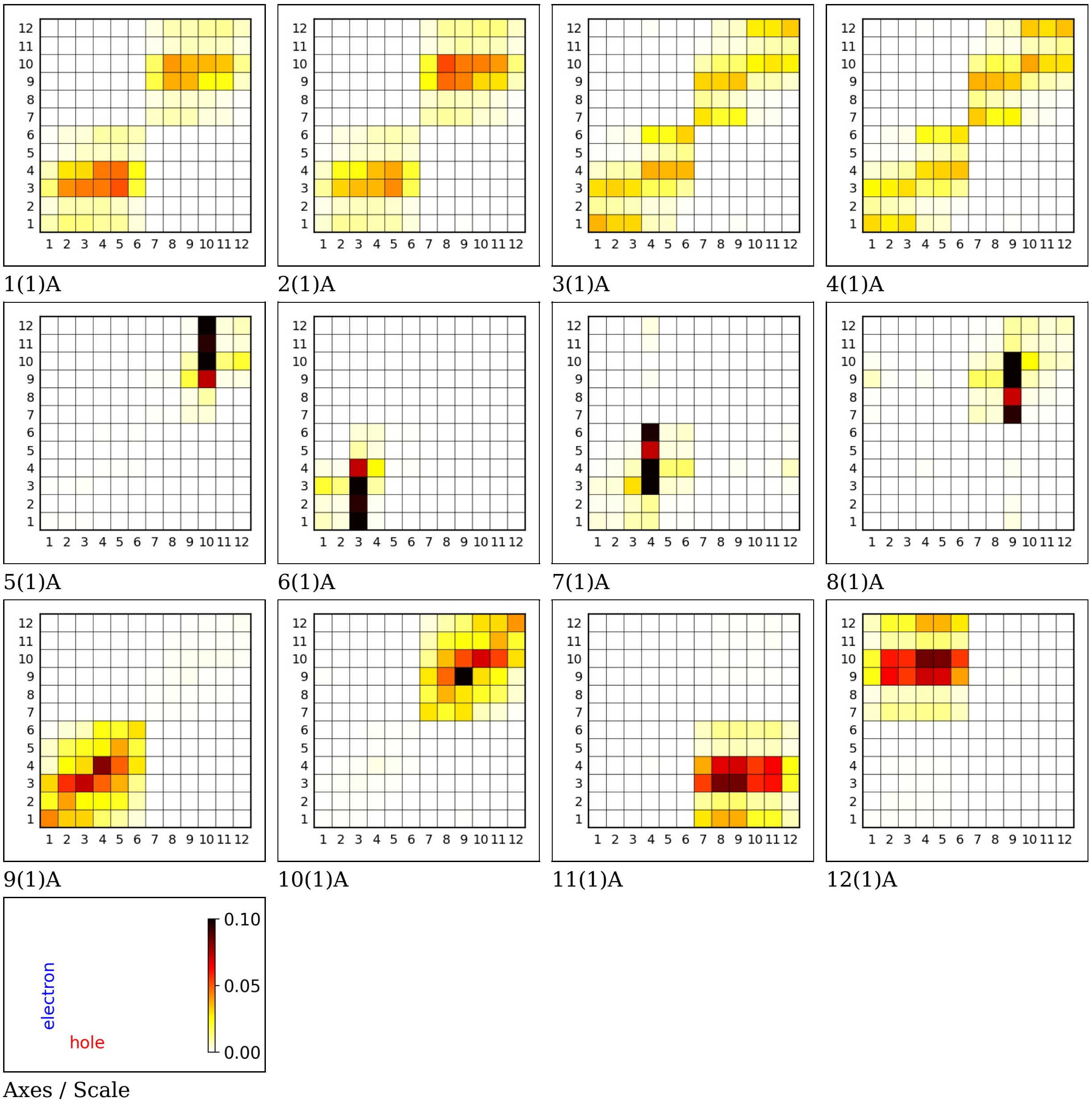

Figure S8: βQA **H-Bond** Ω **matrices excitation analysis: dimer (embedded) .** βQA gas phase dimer electron-hole correlation analysis represented as Ω matrices as obtained through TheoDORE[3] with with def2-TZVP basis set and employing 3 layers of point-charges around the molecule.. Excited states (1A, 2A, etc.) ordered from top left by rows. The colorscale is reported in the last box.

State	Energy (eV)	Wavelength (nm)	Oscillator Strength	μ_x(au)	μ_y(au)	μ_z(au)
1	3.39819	364.9	0.000000006	-0.00021	0.00014	0.00006
2	3.50325	353.9	0.294780438	-1.46607	1.05865	0.40553
3	3.82699	324.0	0.000000000	-0.00000	0.00006	0.00004
4	3.82871	323.8	0.000014054	-0.00401	-0.00668	-0.00944
5	3.86906	320.5	0.000583918	-0.05525	0.04867	0.02720
6	3.87101	320.3	0.000000011	0.00023	-0.00021	-0.00013
7	4.0655	305.0	0.000000002	0.00009	-0.00006	0.00012
8	4.07093	304.6	0.005719503	-0.17569	0.11679	-0.11331
9	4.26933	290.4	0.002935783	0.01425	0.03180	0.16387
10	4.33146	286.2	0.000000000	0.00000	-0.00000	0.00001
11	4.42026	280.5	0.000000025	-0.00014	0.00014	0.00043
12	4.42982	279.9	0.090254278	-0.34519	0.30097	0.78860

Table S6: β**QA** π**-stacked monodimensional aggregate: dimer (vacuum).** Results for the βQA gas-phase dimer obtained with TDDFT at ωB97X with def2-TZVP basis set. Rows marked in green correspond to the selected electronic states possessing excitonic features.

State	Energy (eV)	Wavelength (nm)	Oscillator Strength	μ_x(au)	μ_y(au)	μ_z(au)
1	3.20991	386.3	0.000001079	0.00265	-0.00191	-0.00176
2	3.31774	373.7	0.303420197	-1.52191	1.09838	0.45853
3	3.84988	322.0	0.000922684	0.07979	-0.05536	0.01873
4	3.86386	320.9	0.009288988	-0.24989	0.17699	-0.06603
5	4.0921	303.0	0.000041995	-0.00521	0.01213	0.01564
6	4.09789	302.6	0.000064844	0.00485	-0.01380	-0.02078
7	4.12985	300.2	0.004356302	0.01657	0.02606	0.20519
8	4.14175	299.4	0.000385288	-0.02254	0.02443	0.05189
9	4.14447	299.2	0.000247708	-0.01933	0.01977	0.04093
10	4.19876	295.3	0.000004378	0.00265	-0.00245	-0.00544
11	4.29941	288.4	0.000007729	0.00213	-0.00262	-0.00787
12	4.31398	287.4	0.075798414	0.35439	-0.29897	-0.70866

Table S7: β**QA** π**-stacked monodimensional aggregate: dimer (embedded).** Results for the βQA embedded dimer obtained with TDDFT at ωB97X with def2-TZVP basis set and employing 3 layers of point-charges around the molecule. Rows marked in green correspond to the selected electronic states possessing excitonic features.

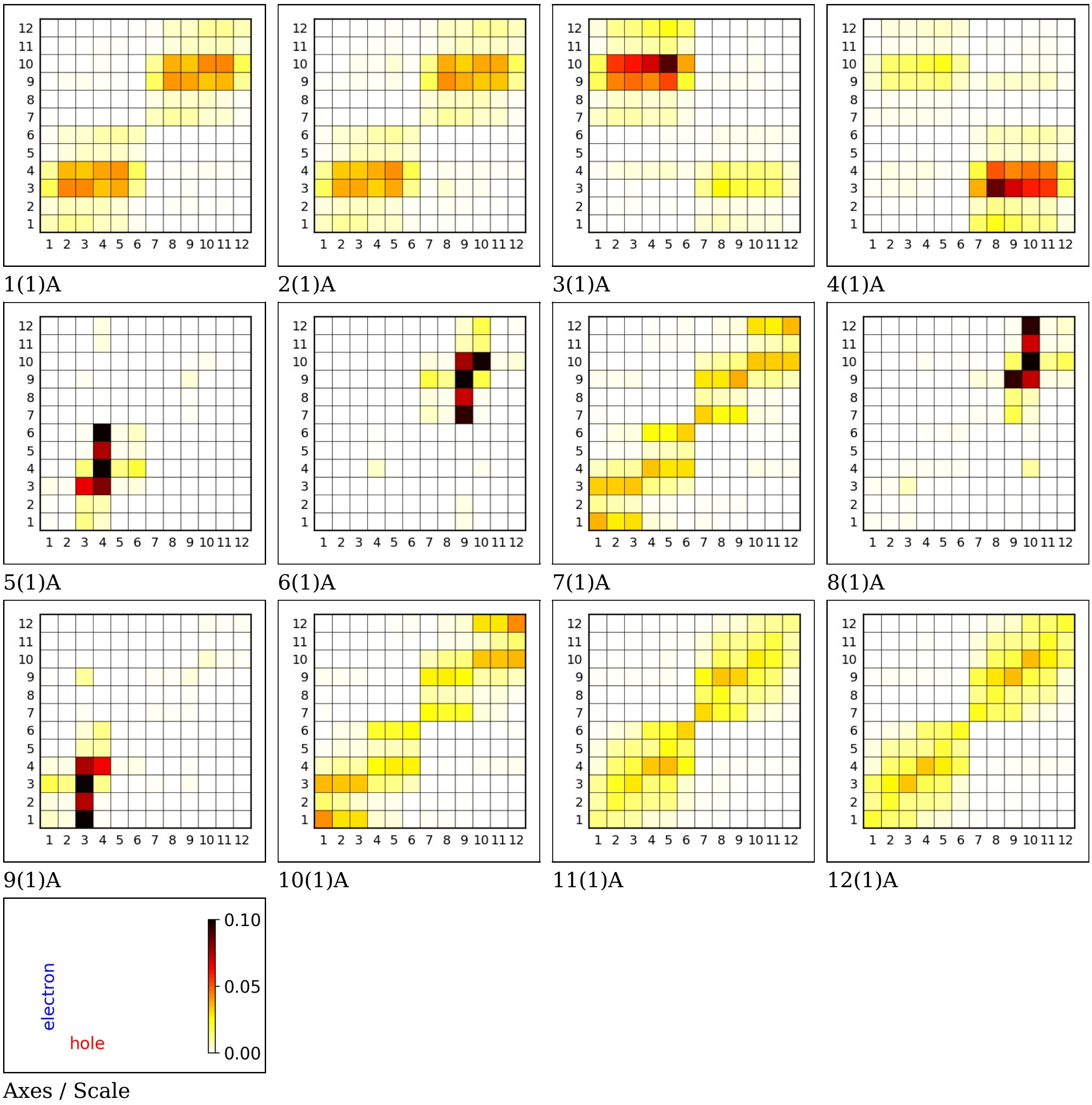

Figure S9: β**QA** π**-stacked** Ω **matrices excitation analysis: dimer (embedded) .** βQA embedded dimer electron-hole correlation analysis represented as Ω matrices as obtained from `TheoDORE`[3] with TDDFT at ωB97X with def2-TZVP basis set. Excited states (1A, 2A, etc.) ordered from top left by rows. The colorscale is defined in the last box. Corresponding transition energies and dipole moments listed in table S7.

State	Energy (eV)	Wavelength (nm)	Oscillator Strength	μ_x(au)	μ_y(au)	μ_z(au)
1	3.32872	372.5	0.443340057	0.79344	0.04895	-2.19189
2	3.39125	365.6	0.023808317	0.00370	-0.51731	0.13761
3	3.78501	327.6	0.000078057	0.02079	0.01991	-0.00365
4	3.83644	323.2	0.000006829	0.00485	-0.00681	0.00166
5	3.87599	319.9	0.000128342	-0.02871	0.02287	0.00204
6	4.06124	305.3	0.000979200	-0.00163	0.02233	-0.09664
7	4.21854	293.9	0.004468782	0.06028	0.12187	-0.15733
8	4.22712	293.3	0.000909010	0.02918	0.05871	-0.06692
9	4.26165	290.9	0.006901507	0.17274	-0.09876	0.16281
10	4.35646	284.6	0.087717747	-0.76077	0.47538	0.13078
11	4.38247	282.9	0.040947969	0.49873	0.27528	-0.23847
12	4.98476	248.7	0.479448401	0.90736	-1.53775	0.85903

Table S8: γ**QA H-Bond monodimensional aggregate: dimer (vacuum).** Results for the βQA gas-phase dimer obtained with TDDFT at ωB97X with def2-TZVP basis set. Rows marked in green correspond to the selected electronic states possessing excitonic features.

State	Energy (eV)	Wavelength (nm)	Oscillator Strength	μ_x(au)	μ_y(au)	μ_z(au)
1	3.21911	385.2	0.461103949	-0.81007	-0.05235	2.27765
2	3.28817	377.1	0.024222186	-0.00899	0.53343	-0.12669
3	4.12292	300.7	0.003545342	-0.01283	-0.10811	0.15247
4	4.13769	299.6	0.000244743	-0.03529	-0.03088	0.01468
5	4.14998	298.8	0.000286338	-0.02226	0.02519	-0.04106
6	4.1731	297.1	0.004007037	-0.16282	0.02982	-0.10859
7	4.18497	296.3	0.000250206	-0.04106	0.01405	-0.02360
8	4.26126	291.0	0.000398715	0.00724	0.03269	-0.05194
9	4.27993	289.7	0.044747757	-0.52607	0.32831	0.20548
10	4.29156	288.9	0.034916802	0.43397	0.25545	-0.28020
11	4.39446	282.1	0.000717994	0.00396	-0.02825	0.07652
12	4.98742	248.6	0.000944376	0.03284	-0.02627	0.07720

Table S9: γ**QA H-Bond monodimensional aggregate: dimer (embedded).** Results for the γQA embedded dimer obtained with TDDFT at ωB97X with def2-TZVP basis set and employing 3 layers of point-charges around the molecule. Rows marked in green correspond to the selected electronic states possessing excitonic features.

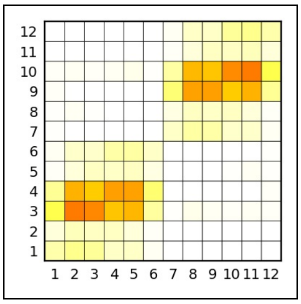

1(1)A

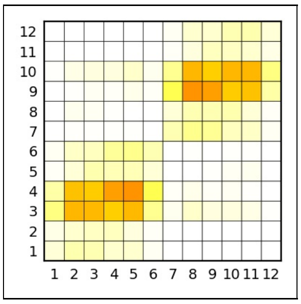

2(1)A

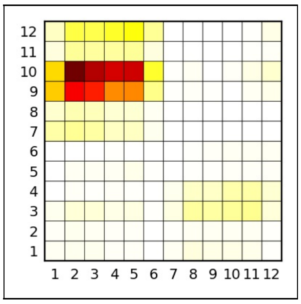

3(1)A

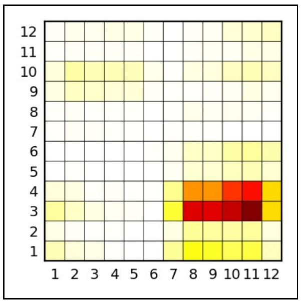

4(1)A

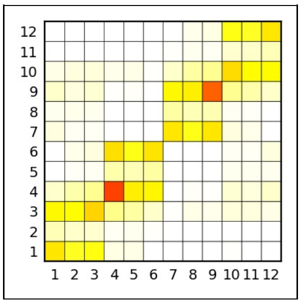

5(1)A

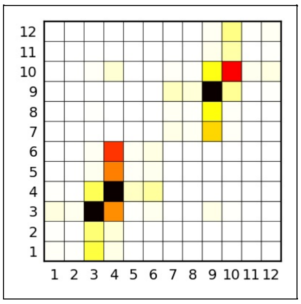

6(1)A

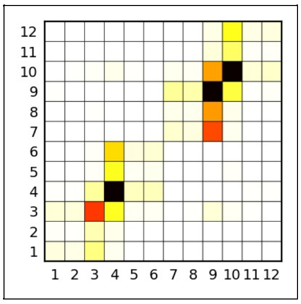

7(1)A

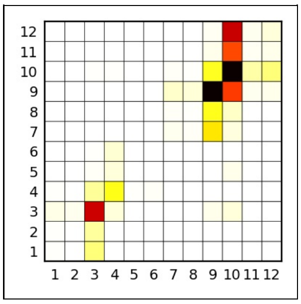

8(1)A

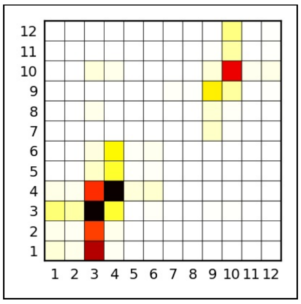

9(1)A

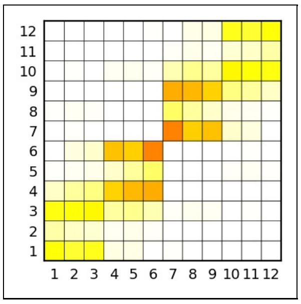

10(1)A

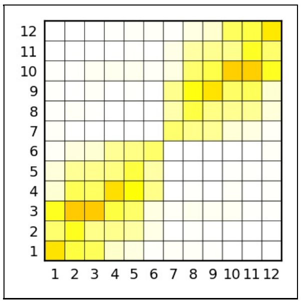

11(1)A

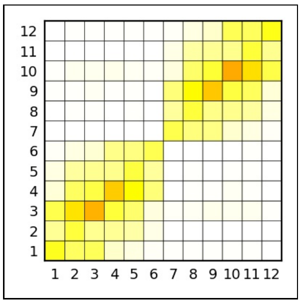

12(1)A

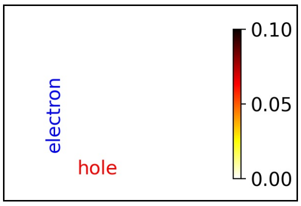

Axes / Scale

Figure S10: γ**QA H-Bond Ω matrices excitation analysis: dimer (embedded) .** γQA embedded dimer electron-hole correlation analysis represented as Ω matrices as obtained from TheoDORE[3] with TDDFT at ωB97X with def2-TZVP basis set. Excited states (1A, 2A, etc.) ordered from top left by rows. The colorscale is defined in the last box. Corresponding transition energies and dipole moments listed in table S9

State	Energy (eV)	Wavelength (nm)	Oscillator Strength	μ_x(au)	μ_y(au)	μ_z(au)
1	3.346	370.5	0.000000036	0.00015	-0.00021	-0.00061
2	3.50375	353.9	0.254968727	0.54637	-0.41563	-1.58083
3	3.7894	327.2	0.000113258	0.02168	-0.02106	-0.01750
4	3.78966	327.2	0.000000004	-0.00009	0.00004	0.00019
5	3.84359	322.6	0.000181713	0.03213	-0.02935	0.00599
6	3.84382	322.6	0.000001415	0.00280	-0.00262	0.00057
7	3.95411	313.6	0.016357749	0.33673	-0.15055	-0.18112
8	3.96065	313.0	0.000064464	0.02109	-0.00946	-0.01141
9	4.25217	291.6	0.004736467	-0.17461	0.12209	0.00852
10	4.33573	286.0	0.000000067	0.00074	0.00012	-0.00027
11	4.39095	282.4	0.000000047	0.00054	-0.00034	-0.00019
12	4.41682	280.7	0.083800787	-0.70492	0.46740	0.24303

Table S10: γ**QA** π**-stacked monodimensional aggregate: dimer (vacuum).** Results for the γQA gas-phase dimer obtained with TDDFT at ωB97X with def2-TZVP basis set. Rows marked in green correspond to the selected electronic states possessing excitonic features.

State	Energy (eV)	Wavelength (nm)	Oscillator Strength	μ_x(au)	μ_y(au)	μ_z(au)
1	3.16156	392.2	0.000000056	-0.00023	0.00020	0.00080
2	3.32858	372.5	0.262155584	-0.58488	0.44320	1.63591
3	3.73429	332.0	0.003851017	-0.12748	0.06611	0.14653
4	3.74246	331.3	0.015695600	-0.25950	0.13411	0.29302
5	4.10743	301.9	0.002596576	-0.12251	0.10303	0.01335
6	4.11611	301.2	0.000010705	0.00852	-0.00567	-0.00121
7	4.11803	301.1	0.000285130	0.04526	-0.02731	-0.00565
8	4.14684	299.0	0.000151783	-0.03010	0.01914	-0.01489
9	4.14737	298.9	0.000024220	-0.01234	0.00768	-0.00520
10	4.20577	294.8	0.000000069	-0.00042	0.00066	0.00025
11	4.27877	289.8	0.000002870	0.00391	-0.00297	-0.00180
12	4.29706	288.5	0.052487490	-0.52116	0.37291	0.29648

Table S11: γ**QA** π**-stacked monodimensional aggregate: dimer (embedded).** Results for the γQA embedded dimer obtained with TDDFT at ωB97X with def2-TZVP basis set and employing 3 layers of point-charges around the molecule. Rows marked in green correspond to the selected electronic states possessing excitonic features.

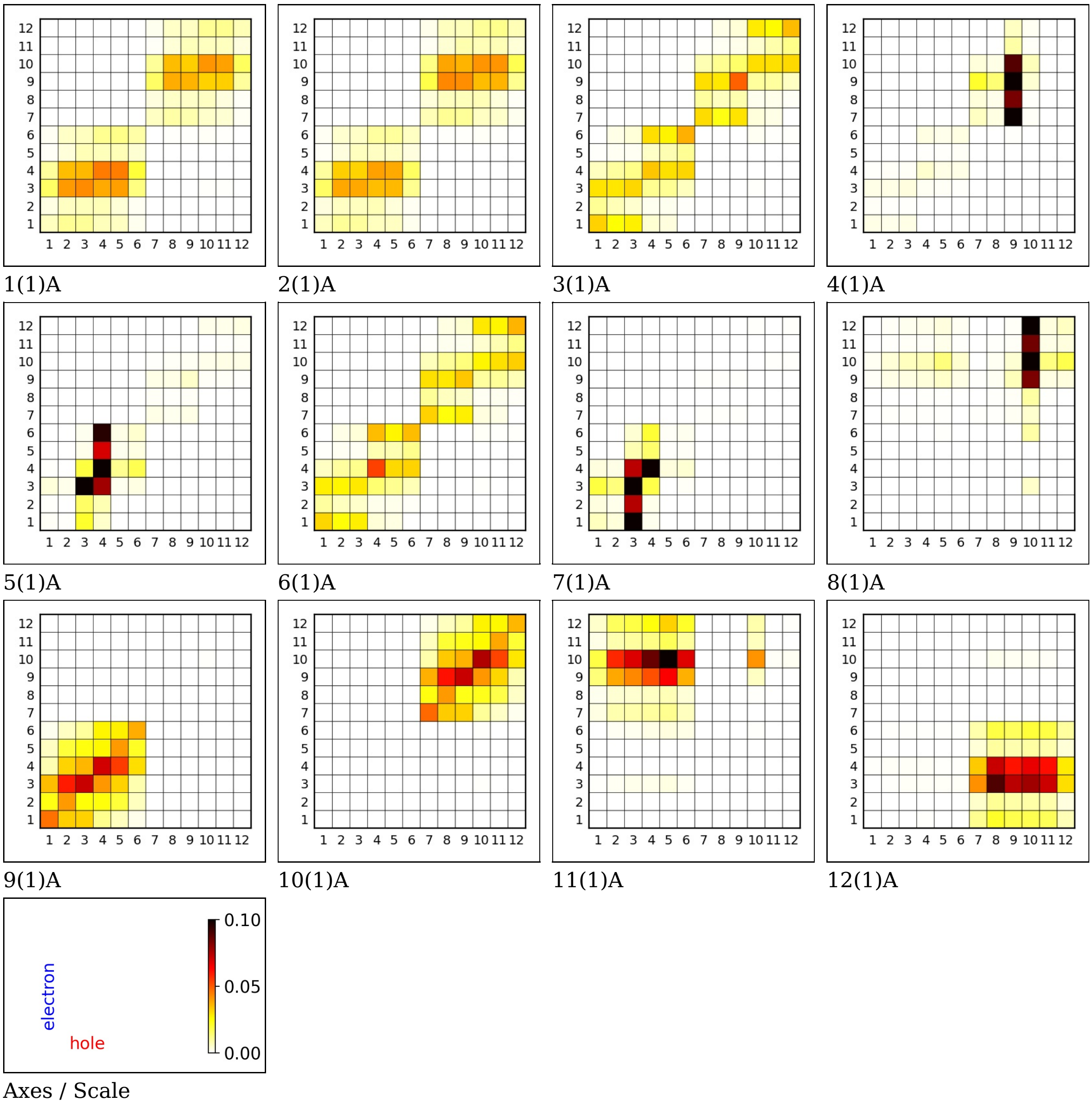

Figure S11: γ**QA** π**-stacked** Ω **matrices excitation analysis: dimer (embedded) .** γQA embedded dimer electron-hole correlation analysis represented as Ω matrices as obtained from `TheoDORE`[3] with TDDFT at ωB97X with def2-TZVP basis set. Excited states (1A, 2A, etc.) ordered from top left by rows. The colorscale is defined in the last box. Corresponding transition energies and dipole moments listed in table S11.

2.3 Hexamers

State	Energy (eV)	Wavelength (nm)	Oscillator Strength	μ_x(au)	μ_y(au)	μ_z(au)
1	3.12036	397.3	1.700501399	3.96180	-2.49057	-0.58764
2	3.183	389.5	0.000000000	0.00002	-0.00001	0.00001
3	3.23101	383.7	0.117669412	-1.01497	0.63909	0.21888
4	3.26323	379.9	0.000000000	-0.00003	0.00002	0.00003
5	3.29274	376.5	0.105529747	0.94328	-0.59507	-0.25353
6	3.29397	376.4	0.000007856	0.00814	-0.00514	-0.00216
7	3.80542	325.8	0.000097612	-0.01347	0.01803	0.02325
8	3.80543	325.8	0.000090460	-0.01293	0.01736	0.02240
9	4.03302	307.4	0.000000002	0.00011	-0.00005	0.00001
10	4.07182	304.5	0.001423097	0.08147	-0.04200	0.07658
11	4.12091	300.9	0.000000000	-0.00001	-0.00000	-0.00002
12	4.16816	297.5	0.002651132	0.09937	-0.04810	0.11736
13	4.20052	295.2	0.000006795	0.00656	-0.00447	0.00173
14	4.20055	295.2	0.000144517	0.03025	-0.02061	0.00803
15	4.21483	294.2	0.000000037	-0.00041	0.00019	-0.00040
16	4.21815	293.9	0.005596338	0.14456	-0.07047	0.16820
17	4.21871	293.9	0.000030967	-0.01033	0.00492	-0.01299
18	4.22204	293.7	0.002159156	0.05417	-0.01636	0.13294

Table S12: β**QA H-Bond monodimensional aggregate: hexamer (vacuum).** Results for the βQA gas-phase hexamer obtained with TDDFT at ωB97X with def2-TZVP basis set. Rows marked in green correspond to the selected electronic states possessing excitonic features.

State	Energy (eV)	Wavelength (nm)	Oscillator Strength	μ_x(au)	μ_y(au)	μ_z(au)
1	2.98084	415.9	0.739952469	2.66074	-1.67610	-0.49339
2	2.99371	414.2	0.240360110	-1.51382	0.95348	0.27637
3	3.09924	400.0	0.769191155	-2.67648	1.69046	0.33034
4	3.13439	395.6	0.001106024	0.10103	-0.06382	-0.01108
5	3.19802	387.7	0.042911214	-0.62354	0.39216	0.07139
6	3.25357	381.1	0.000000204	-0.00135	0.00084	0.00017
7	3.98838	310.9	0.006912707	-0.03744	0.03512	0.26098
8	3.99483	310.4	0.053242517	-0.10038	0.09455	0.72456
9	4.0256	308.0	0.001104622	-0.00281	0.00755	0.10552
10	4.02842	307.8	0.002283174	0.00098	-0.00906	-0.15183
11	4.04176	306.8	0.000010998	-0.00140	0.00156	0.01033
12	4.07065	304.6	0.012965191	-0.01360	0.01847	0.35983
13	4.10386	302.1	0.000596476	-0.00380	-0.00948	-0.07634
14	4.10828	301.8	0.000647490	-0.00540	-0.00977	-0.07943
15	4.11643	301.2	0.000014260	-0.00033	-0.00073	-0.01186
16	4.16481	297.7	0.049032992	0.08585	-0.09865	-0.68077
17	4.17032	297.3	0.026196665	-0.07485	0.07849	0.49461
18	4.17301	297.1	0.016550912	-0.03549	0.04981	0.39768

Table S13: β**QA H-Bond monodimensional aggregate: hexamer (embedded).** Results for the βQA embedded hexamer obtained with TDDFT at ωB97X with def2-TZVP basis set and employing 3 layers of point-charges around the molecule. Rows marked in green correspond to the selected electronic states possessing excitonic features.

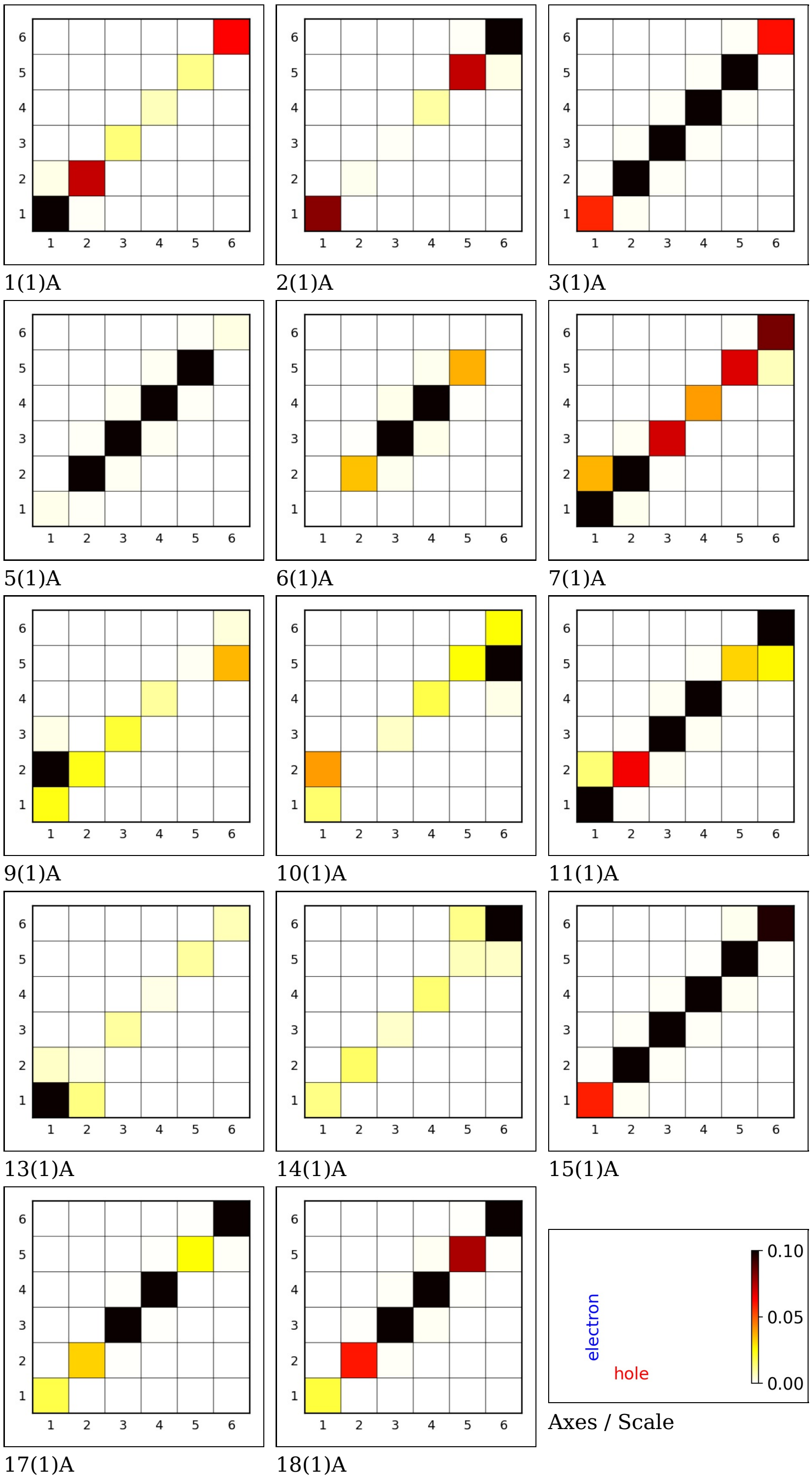

Figure S12: γ**QA H-Bond Ω matrices excitation analysis: hexamer (embedded) .** βQA embedded dimer electron-hole correlation analysis represented as Ω matrices as obtained from `TheoDORE`[3] with TDDFT at ωB97X with def2-TZVP basis set. Excited states (1A, 2A, etc.) ordered from top left by rows. The colorscale is defined in the last box. Corresponding transition energies and dipole moments listed in table S13.

State	Energy (eV)	Wavelength (nm)	Oscillator Strength	μ_x(au)	μ_y(au)	μ_z(au)
1	3.36786	368.1	0.000000002	-0.00012	0.00007	0.00004
2	3.3891	365.8	0.018094111	0.38549	-0.24351	-0.10010
3	3.42519	362.0	0.000000001	0.00008	-0.00005	-0.00003
4	3.47	357.3	0.075186365	0.75244	-0.52543	-0.20535
5	3.51342	352.9	0.000000005	0.00017	-0.00017	-0.00003
6	3.54315	349.9	0.672298506	2.02169	-1.84124	-0.51721
7	3.82486	324.2	0.000000000	0.00003	0.00000	0.00003
8	3.82571	324.1	0.000000397	-0.00157	0.00103	0.00084
9	3.82677	324.0	0.000000000	-0.00002	-0.00000	-0.00005
10	3.82774	323.9	0.000004282	0.00219	0.00491	0.00409
11	3.82847	323.8	0.000000001	-0.00002	0.00003	-0.00008
12	3.8287	323.8	0.000004196	0.00298	0.00398	0.00447
13	3.86006	321.2	0.001124744	0.06905	-0.08226	-0.01893
14	3.86124	321.1	0.000000000	-0.00003	0.00000	-0.00005
15	3.86264	321.0	0.000119527	0.02425	-0.02485	-0.00760
16	3.86382	320.9	0.000000000	-0.00002	0.00004	-0.00002
17	3.86988	320.4	0.000499862	0.04538	-0.05004	-0.02664
18	3.86998	320.4	0.000001556	-0.00255	0.00285	0.00133

Table S14: β**QA** π**-stacked monodimensional aggregate: hexamer (vacuum).** Results for the βQA gas-phase hexamer obtained with TDDFT at ωB97X with def2-TZVP basis set and employing 3 layers of point-charges around the molecule. Rows marked in green correspond to the selected electronic states possessing excitonic features.

State	Energy (eV)	Wavelength (nm)	Oscillator Strength	μ_x(au)	μ_y(au)	μ_z(au)
1	3.15303	393.2	0.000000005	0.00005	-0.00007	-0.00023
2	3.1813	389.7	0.004460019	-0.20823	0.10608	0.05109
3	3.22315	384.7	0.000000296	0.00135	-0.00107	-0.00088
4	3.2704	379.1	0.030941792	0.52273	-0.29877	-0.15384
5	3.31129	374.4	0.000000502	0.00210	-0.00130	-0.00030
6	3.33315	372.0	0.752660546	-2.21086	1.98301	0.62991
7	3.83107	323.6	0.000314230	0.04502	-0.02873	0.02227
8	3.8336	323.4	0.001429573	-0.09437	0.06342	-0.04788
9	3.84644	322.3	0.000300603	-0.03348	0.01760	-0.04195
10	3.84772	322.2	0.000062713	0.01542	-0.00766	0.01921
11	3.86695	320.6	0.000089670	0.02239	-0.02044	0.00524
12	3.8707	320.3	0.003275203	-0.13880	0.11830	-0.03574
13	3.88466	319.2	0.000083607	0.01361	-0.00106	-0.02631
14	3.89094	318.7	0.000010001	-0.00571	0.00746	-0.00408
15	3.89914	318.0	0.000002289	-0.00165	0.00426	-0.00175
16	3.90902	317.2	0.029900360	0.35682	-0.41832	0.09953
17	4.08551	303.5	0.000320744	-0.00210	-0.00964	-0.05574
18	4.10571	302.0	0.000001143	-0.00041	0.00076	0.00326

Table S15: β**QA** π**-stacked monodimensional aggregate: hexamer (embedded).** Results for the βQA embedded hexamer obtained with TDDFT at ωB97X with def2-TZVP basis set and employing 3 layers of point-charges around the molecule. Rows marked in green correspond to the selected electronic states possessing excitonic features.

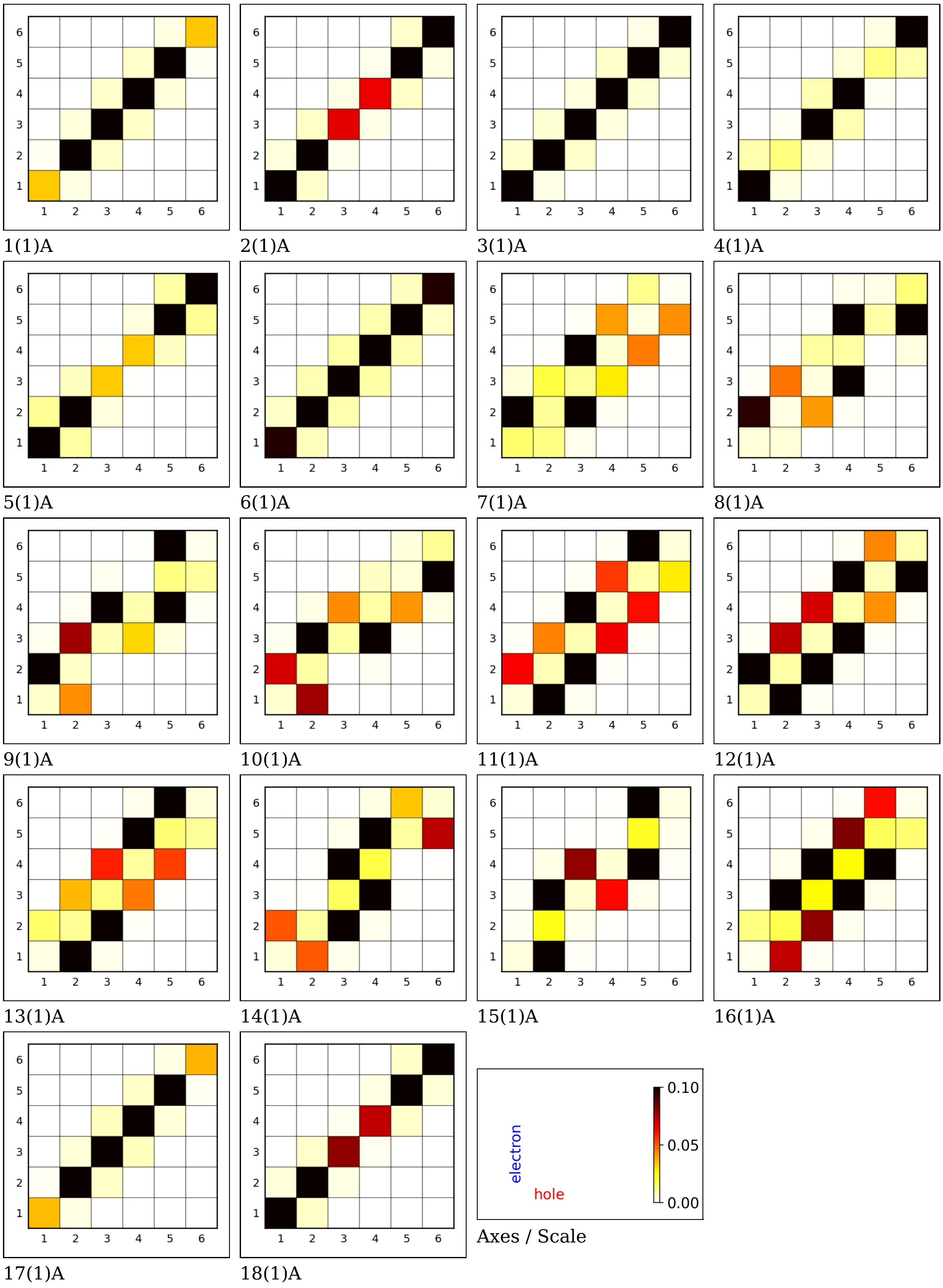

Figure S13: β**QA** π**-stacked** Ω **matrices excitation analysis: hexamer (embedded) .** βQA embedded hexamer electron-hole correlation analysis represented as Ω matrices as obtained from `TheoDORE`[3] with TDDFT at ωB97X with def2-TZVP basis set. Excited states (1A, 2A, etc.) ordered from top left by rows. The colorscale is defined in the last box. Corresponding transition energies and dipole moments listed in table S15.

State	Energy (eV)	Wavelength (nm)	Oscillator Strength	μ_x(au)	μ_y(au)	μ_z(au)
1	3.2097	386.3	1.494468804	1.29117	0.01849	-4.16383
2	3.26595	379.6	0.000114830	-0.02900	-0.01738	0.01710
3	3.30954	374.6	0.112566966	-0.39381	-0.01910	1.11034
4	3.33977	371.2	0.008390210	-0.03094	-0.31826	0.01727
5	3.36525	368.4	0.078934804	0.33325	-0.38485	-0.83561
6	3.36883	368.0	0.072909711	0.17926	0.71430	-0.58397
7	3.77042	328.8	0.000075317	0.02034	0.01980	-0.00309
8	3.80533	325.8	0.000083095	0.02262	-0.01923	-0.00312
9	3.81376	325.1	0.000075667	0.02126	0.01859	-0.00351
10	3.8147	325.0	0.000077673	-0.02166	0.01856	0.00418
11	3.82278	324.3	0.000075324	-0.02135	-0.01814	0.00441
12	3.84145	322.8	0.000004785	0.00150	-0.00690	0.00097
13	3.87986	319.6	0.000129829	-0.02983	0.02179	0.00107
14	4.12994	300.2	0.025342656	0.10659	0.02549	-0.48832
15	4.15486	298.4	0.000868888	0.06850	0.01814	0.05929
16	4.18179	296.5	0.002262748	0.04981	0.04817	-0.13147
17	4.20263	295.0	0.003685260	-0.05684	-0.17588	-0.04036
18	4.25018	291.7	0.000254292	0.02128	-0.02900	0.03389

Table S16: γ**QA H-Bond monodimensional aggregate: hexamer (vacuum).** Results for the βQA gas-phase hexamer obtained with TDDFT at ωB97X with def2-TZVP basis set. Rows marked in green correspond to the selected electronic states possessing excitonic features.

State	Energy (eV)	Wavelength (nm)	Oscillator Strength	μ_x(au)	μ_y(au)	μ_z(au)
1	3.13411	395.6	1.573583859	1.32995	0.02244	-4.32717
2	3.18658	389.1	0.000149768	-0.03491	0.02611	-0.00421
3	3.22893	384.0	0.107125721	-0.39349	-0.02707	1.09482
4	3.25919	380.4	0.000125414	-0.03353	-0.01126	-0.01788
5	3.27784	378.3	0.046436573	-0.26488	0.14944	0.69696
6	3.28362	377.6	0.066068288	0.04637	0.88006	-0.21122
7	4.0708	304.6	0.020619950	-0.03270	-0.03234	0.45237
8	4.09396	302.8	0.001813854	0.11548	0.00877	0.06836
9	4.12133	300.8	0.001200576	-0.00723	-0.05977	0.09091
10	4.13021	300.2	0.000176600	0.03597	0.02041	-0.00589
11	4.14506	299.1	0.002268234	0.12319	0.01917	0.08242
12	4.15228	298.6	0.000019633	0.00567	-0.01262	0.00126
13	4.15313	298.5	0.000249215	0.01832	-0.02046	0.04117
14	4.15603	298.3	0.000278394	0.04886	-0.00171	0.01854
15	4.1587	298.1	0.000308816	0.04043	0.01303	0.03503
16	4.16059	298.0	0.000201343	0.03946	0.01947	0.00626
17	4.16567	297.6	0.000658401	0.02566	-0.07282	0.02214
18	4.16944	297.4	0.001924279	0.09359	-0.08152	0.05861

Table S17: γ**QA H-Bond monodimensional aggregate: hexamer (embedded).** Results for the γQA embedded hexamer obtained with TDDFT at ωB97X with def2-TZVP basis set and employing 3 layers of point-charges around the molecule. Rows marked in green correspond to the selected electronic states possessing excitonic features.

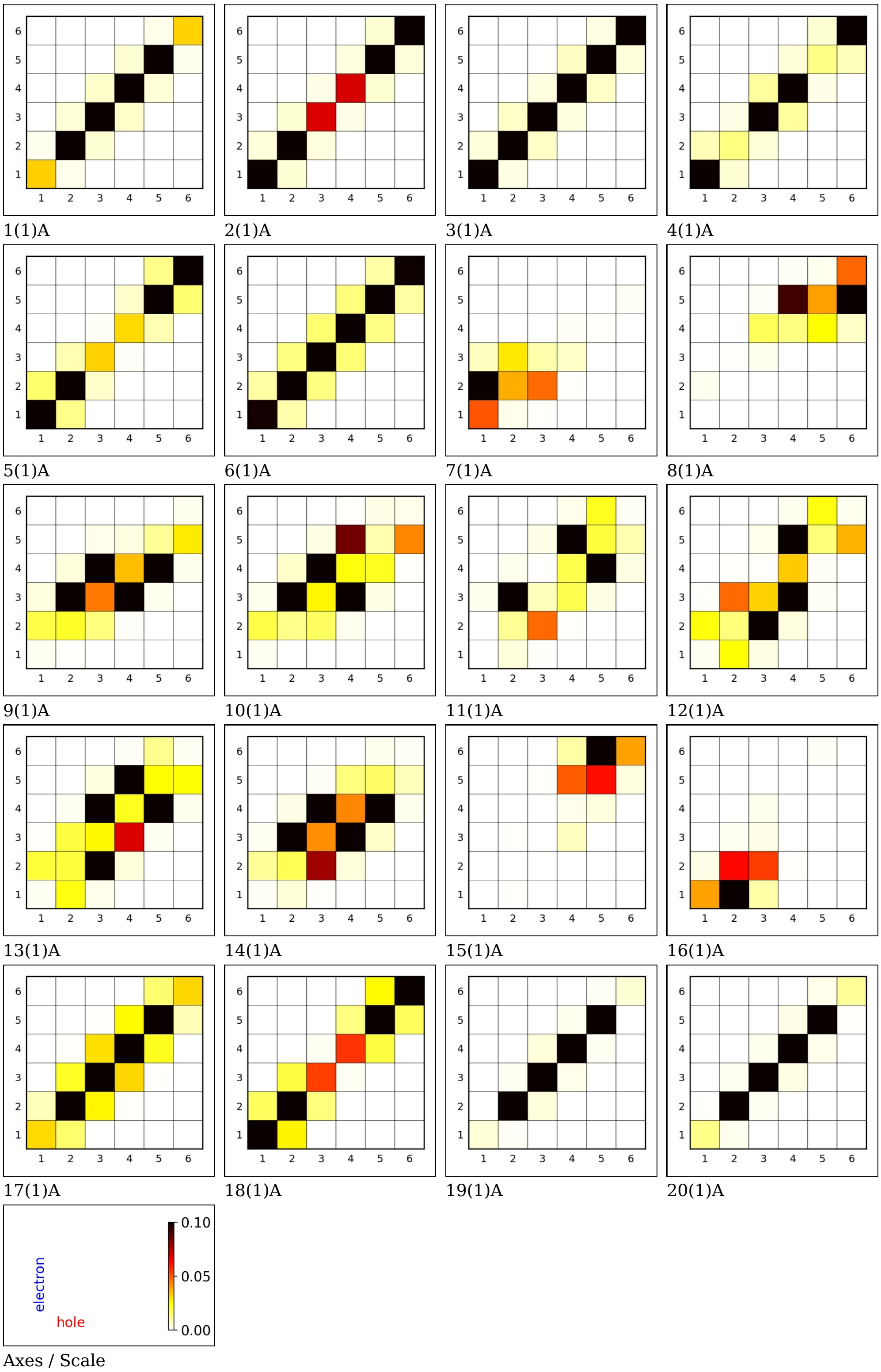

Figure S14: γ**QA H-Bond** Ω **matrices excitation analysis: hexamer (embedded) .** γQA embedded hexamer electron-hole correlation analysis represented as Ω matrices as obtained from `TheoDORE`[3] with TDDFT at ωB97X with def2-TZVP basis set. Excited states (1A, 2A, etc.) ordered from top left by rows. The colorscale is defined in the last box. Corresponding transition energies and dipole moments listed in table S13.

State	Energy (eV)	Wavelength (nm)	Oscillator Strength	μ_x(au)	μ_y(au)	μ_z(au)
1	3.28733	377.2	0.000000005	-0.00007	0.00009	0.00022
2	3.32086	373.4	0.008049508	0.11557	-0.08142	-0.28098
3	3.37362	367.5	0.000000010	-0.00005	0.00012	0.00032
4	3.4381	360.6	0.040592013	-0.23996	0.17063	0.62866
5	3.50038	354.2	0.000000126	-0.00025	0.00036	0.00113
6	3.54411	349.8	0.466558632	-0.65044	0.61750	2.13751
7	3.77955	328.0	0.000000232	-0.00027	-0.00139	-0.00071
8	3.77978	328.0	0.000000002	0.00001	0.00003	0.00014
9	3.78013	328.0	0.000002087	0.00114	-0.00292	-0.00356
10	3.78045	328.0	0.000000010	0.00002	-0.00009	-0.00031
11	3.78925	327.2	0.000083754	-0.01694	0.01885	0.01612
12	3.78926	327.2	0.000077192	-0.01626	0.01806	0.01553
13	3.81251	325.2	0.000590076	-0.05529	0.05708	0.00164
14	3.81256	325.2	0.000000582	-0.00174	0.00178	0.00007
15	3.81271	325.2	0.000014596	-0.00830	0.00934	0.00012
16	3.8128	325.2	0.000000691	0.00186	-0.00198	-0.00008
17	3.84472	322.5	0.000100863	0.02209	-0.02213	0.00967
18	3.84473	322.5	0.000045547	-0.01442	0.01487	-0.00737

Table S18: γ**QA** π**-stacked monodimensional aggregate: hexamer (vacuum).** Results for the γQA gas-phase hexamer obtained with TDDFT at ωB97X with def2-TZVP basis set. Rows marked in green correspond to the selected electronic states possessing excitonic features.

State	Energy (eV)	Wavelength (nm)	Oscillator Strength	μ_x(au)	μ_y(au)	μ_z(au)
1	3.08224	402.3	0.000000003	0.00005	-0.00005	-0.00017
2	3.12082	397.3	0.004877294	0.09179	-0.05986	-0.22756
3	3.17983	389.9	0.000000017	0.00010	-0.00011	-0.00045
4	3.25015	381.5	0.031135518	-0.22131	0.14573	0.56640
5	3.31617	373.9	0.000000899	0.00083	-0.00091	-0.00309
6	3.35948	369.1	0.492767625	-0.73502	0.68768	2.23022
7	3.696	335.5	0.003806643	-0.12080	0.07134	0.14952
8	3.70169	334.9	0.002439649	0.09822	-0.05672	-0.11848
9	3.71998	333.3	0.000980486	0.06423	-0.03826	-0.07190
10	3.72944	332.4	0.000115760	-0.01974	0.01494	0.02557
11	3.73466	332.0	0.000968397	-0.05864	0.04182	0.07346
12	3.74163	331.4	0.003058747	-0.10721	0.07418	0.12795
13	3.7536	330.3	0.000922476	0.05909	-0.04129	-0.06953
14	3.76073	329.7	0.045584402	0.41578	-0.29363	-0.48545
15	3.77136	328.8	0.008837951	0.18774	-0.11777	-0.21573
16	3.77809	328.2	0.011867586	0.21870	-0.13832	-0.24749
17	4.07396	304.3	0.000224846	0.03771	-0.02874	-0.00223
18	4.09114	303.1	0.000000146	-0.00034	0.00088	-0.00075

Table S19: γ**QA** π**-stacked monodimensional aggregate: hexamer (embedded).** Results for the γQA embedded hexamer obtained with TDDFT at ωB97X with def2-TZVP basis set and employing 3 layers of point-charges around the molecule. Rows marked in green correspond to the selected electronic states possessing excitonic features.

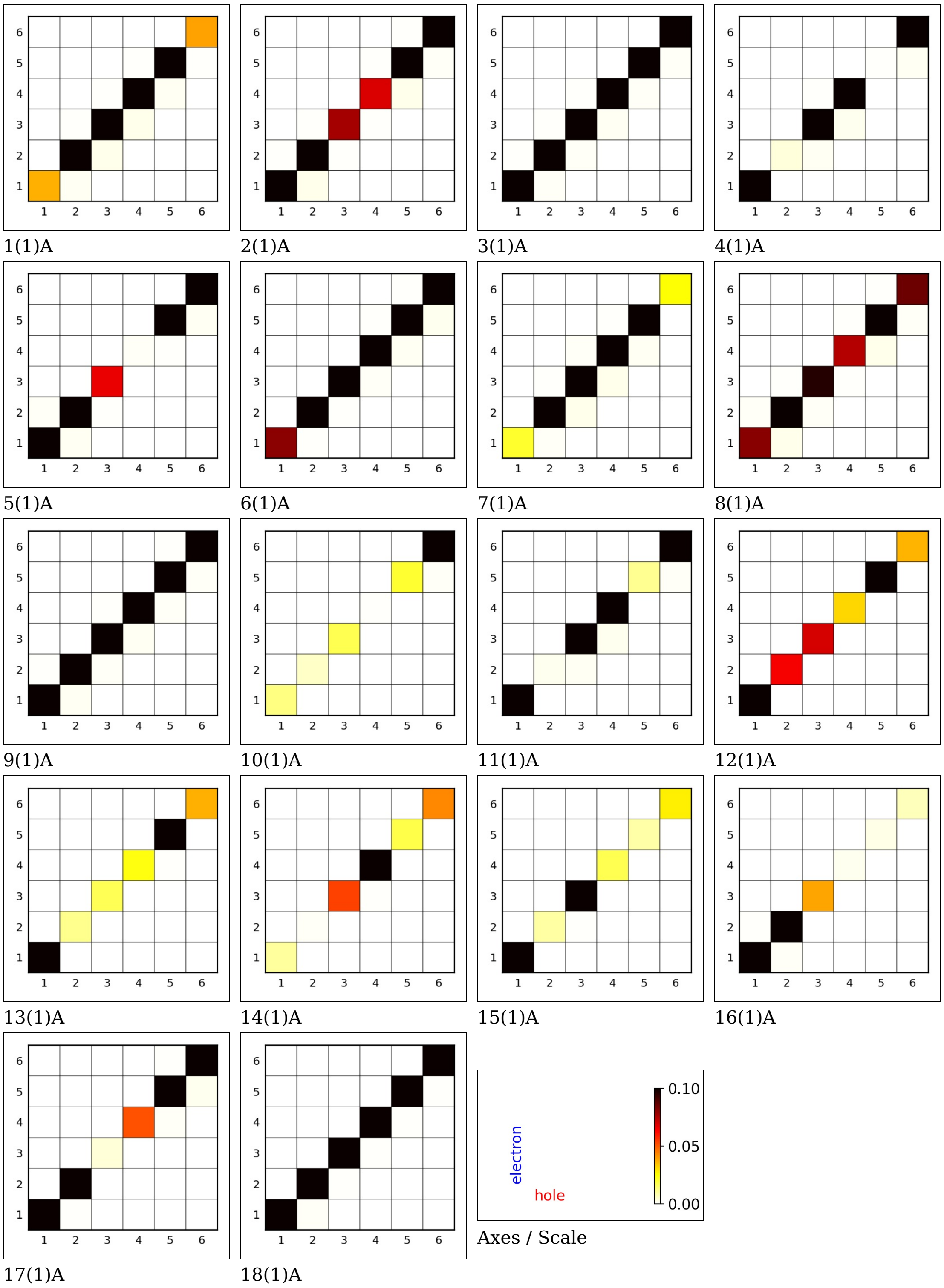

Figure S15: **βQA π-stacked Ω matrices excitation analysis: hexamer (embedded) .** βQA embedded hexamer electron-hole correlation analysis represented as Ω matrices as obtained from `TheoDORE`[3] with TDDFT at ωB97X with def2-TZVP basis set. Excited states (1A, 2A, etc.) ordered from top left by rows. The colorscale is defined in the last box. Corresponding transition energies and dipole moments listed in table S19

3 Linear aggregate fitting

To obtain the electronic parameters J for the FE Hamiltonian we adopted a reverse-eigenvalue approach. First, we track the evolution of simulated TD-DFT transitions on monodimensional aggregates of increasing size (up to hexamer), as seen in Fig. S16. Hexamers are then selected, offering a good compromise between accuracy and computational feasibility. For each hexamer aggregate, electron-hole correlation analysis is employed to confirm the excitonic character of the states used to map the model. In the case of γQA $_H$,γQA $_\pi$ and βQA $_\pi$, the first N= 6 excited states are essentially purely excitonic and were safely selected. For βQA $_H$, as also visible in Fig. S12), we find that the two lowest-energy states exhibited poor excitonic character, as they include CT contributions and are rather localised on the two molecules at the ends of the linear cluster. We solved this finite-size effect by selecting the remaining (N-2) states corresponding to excitonic states delocalised on the (N-2) inner molecules. Summarizing, we selected the first six states of γQA $_H$,γQA $_\pi$ and βQA $_\pi$ embedded hexamers against TDDFT results, and the third to the sixth states of the βQA $_H$ embedded hexamers.

Next, we employed an in-house script that by fitting the TD-DFT energies of the selected transitions (as specified above) finds the best values for the diagonal energy ϵ_0 and the excitonic coupling J of a *fitting Hamiltonian* for each aggregate. Namely we employed an open-boundary condition excitonic Hamiltonian: $\hat{H}_{exc} = \sum_i^N \varepsilon \left|i\right\rangle\left\langle i\right| + \sum_i^{N-1} J(\left|i+1\right\rangle\left\langle i\right| + \left|i\right\rangle\left\langle i+1\right|)$ The fitting was performed using `leastsq` least-square algorithm from `scipy python` [4]. Since the diagonal energies are ignored and replaced by an estimate from the experiment, this procedure is only adopted to estimate the excitonic couplings J (Table 1). We note that the oscillator strengths are not actually fitted but their relative magnitude is naturally obtained by the eigenvectors of the fitting Hamiltonian. Actually, the sign of J is fixed by the clustering of the oscillator strength on the bottom or top of the exciton band. Of course, in the exciton model we only get the relative oscillator strengths, and for the sake of comparison in Fig. S17, we have normalised them to the value of the brightest transition.

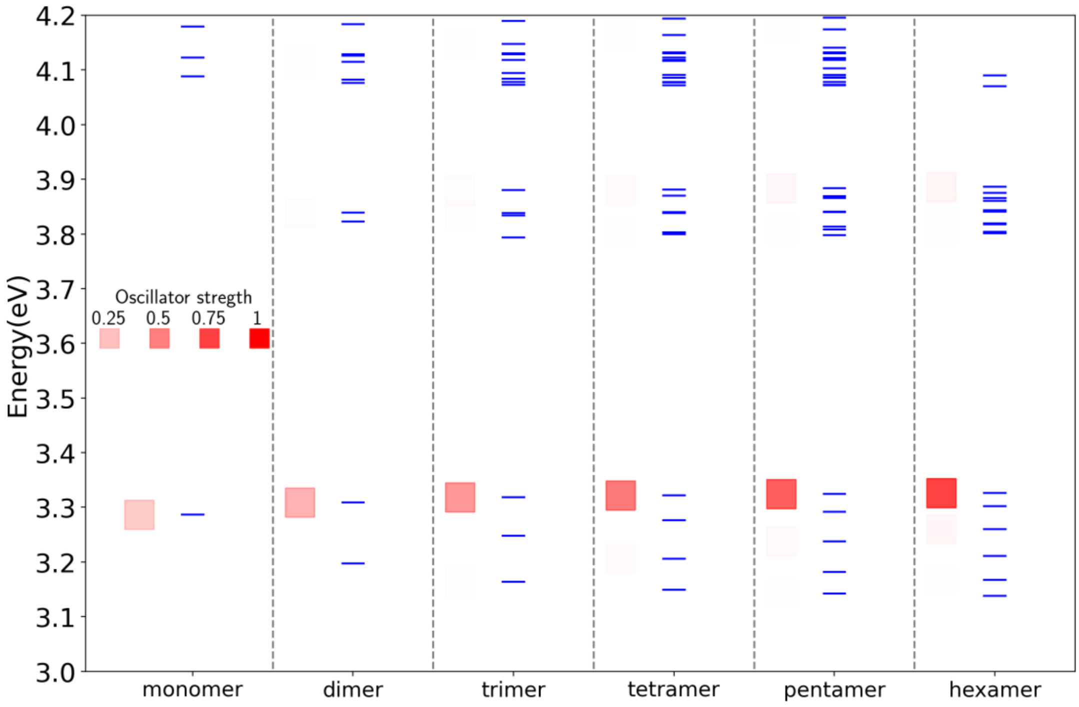

Figure S16: **Monomer to hexamer transitions in the embedded βQA π-stacked aggregates.** TD-DFT results βQA embedded π-stacked aggregates with increasing dimension. While this case is presented here as an example, similar trends were analyzed for all the four linear aggregates.

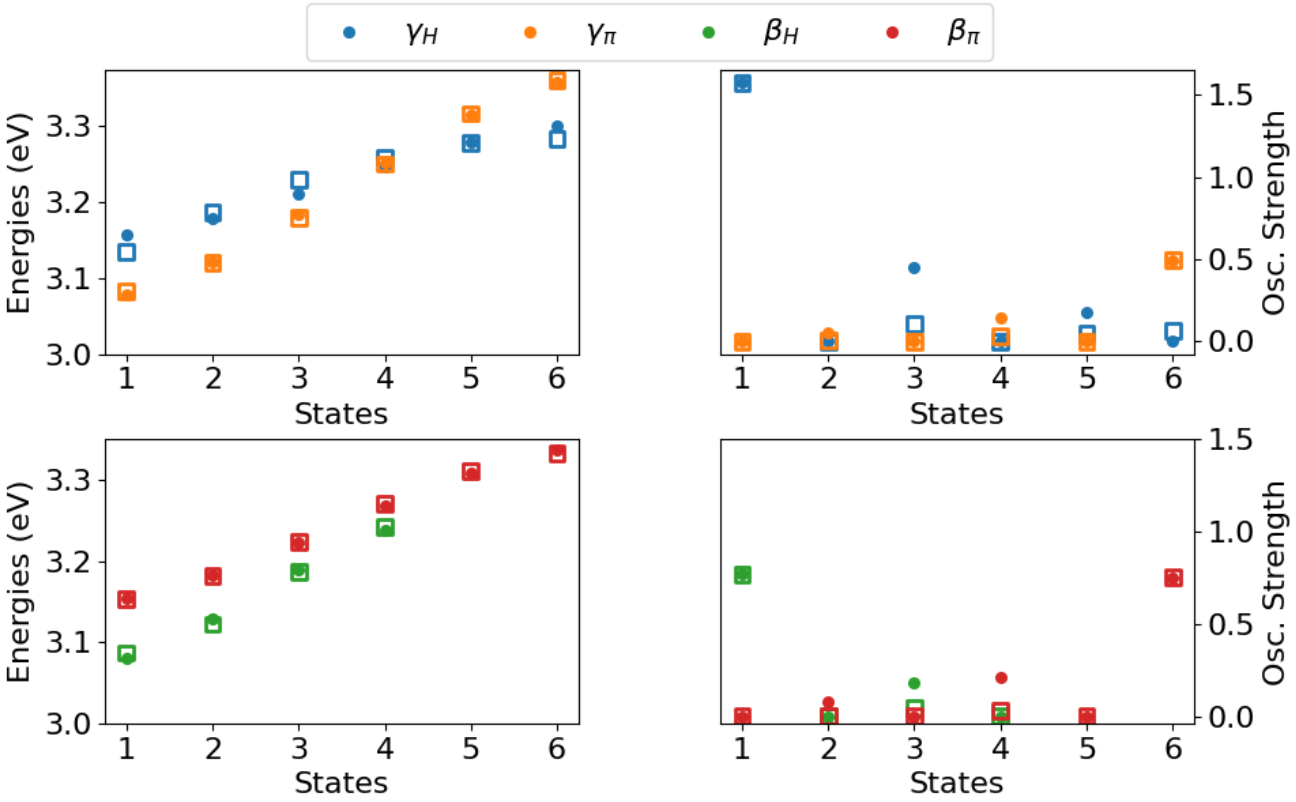

Figure S17: **Fitting of linear aggregates with model Hamiltonian..** Results of the fitting using the model exciton Hamiltonian (circles) against TDDFT results (squares) for the embedded hexamers. On the left: fitted transition energies. On the right: resulting oscillator strength, rescaled to match the brightest transition. Following the oscillator strength trends, γQA $_\pi$,βQA $_\pi$ are H-aggregate while βQA $_H$,γQA $_H$ are J-aggregate. RMS Errors (in meV): γ_H=36.6, γ_π=6.3, β_H=10.1,β_π=5.2

4 Model Hamiltonians

4.1 βQA

βQA can be modeled using a 2D lattice with a single molecule per unit cell. This type of crystal was already employed by Anzola at al. [5, 6], with the only difference that the directions of interaction are not perpendicular to the unit-cell axis. Following a similar analysis the Hamiltonian in the reciprocal space reads:

$$\hat{H}^{\beta} = \sum_{\boldsymbol{k}} [\epsilon_0 + D + 2J^{\beta}_{\pi} cos(\boldsymbol{kb}) + 2J^{\beta}_{H} cos(\boldsymbol{k}(\boldsymbol{a}+\boldsymbol{b}))] \tilde{b}^{\dagger}_{\boldsymbol{k}} \tilde{b}_{\boldsymbol{k}} + \\ + h\omega_{eff} \sum_{\boldsymbol{q}} (\tilde{a}^{\dagger}_{\boldsymbol{q}} \tilde{a}_{\boldsymbol{q}} + \frac{1}{2}) + \frac{g}{\sqrt{N}} \sum_{\boldsymbol{k},\boldsymbol{q}} \tilde{a}^{\dagger}_{\boldsymbol{q}} \tilde{b}^{\dagger}_{\boldsymbol{k}} \tilde{b}_{\boldsymbol{k}+\boldsymbol{q}} + \tilde{a}^{\dagger}_{-\boldsymbol{q}} \tilde{b}^{\dagger}_{\boldsymbol{k}} \tilde{b}_{\boldsymbol{k}+\boldsymbol{q}} \quad (1)$$

and the dipole moment operator:

$$\hat{\boldsymbol{\mu}} = \boldsymbol{\mu_0} \cdot \sqrt{N} (\tilde{b}^{\dagger}_{\boldsymbol{K}=\boldsymbol{0}} + \tilde{b}_{\boldsymbol{K}=\boldsymbol{0}}) \quad (2)$$

4.2 γQA

While the dipole moment operator retains the same formula used for βQA , γQA possesses two molecules per unit cell, and the treatment is more complex. Following Agranovich [7] creation (annihilation) operators in reciprocal space can

be defined. Definining α and β the two molecule in the unit cell and 1 and 2 the two resulting branches, the creation (annihilation) operator for the excitation on the α/β molecule in the $\boldsymbol{n}$ unit cell are written in terms of the operators in the reciprocal space as follows:

$$b^{\dagger}_{\boldsymbol{n}\alpha} = \frac{\sqrt{2}}{2\sqrt{N}} \sum_{\boldsymbol{k}} e^{i\boldsymbol{k}(\boldsymbol{n}+\boldsymbol{r}_{\alpha})}(b^{\dagger}_{1,\boldsymbol{k}} + b^{\dagger}_{2,\boldsymbol{k}}) \tag{3}$$

$$b^{\dagger}_{\boldsymbol{n}\beta} = \frac{\sqrt{2}}{2\sqrt{N}} \sum_{\boldsymbol{k}} e^{i\boldsymbol{k}(\boldsymbol{n}+\boldsymbol{r}_{\beta})}(b^{\dagger}_{1,\boldsymbol{k}} - b^{\dagger}_{2,\boldsymbol{k}}) \tag{4}$$

where $b^{\dagger}_{1,\boldsymbol{k}}, b^{\dagger}_{2,\boldsymbol{k}}$ create an exciton on the first and the second branches respectively. Since all molecules are equivalent by symmetry, the on-site energies for the α and β sites are the same and the Hamiltonian in the real space is:

$$\hat{H} = \sum_{\boldsymbol{i}\sigma} \Big\{ \Big[\epsilon_0 + D - g(\hat{a}^{\dagger}_{\boldsymbol{i}} + \hat{a}_{\boldsymbol{i}})\Big]\hat{b}^{\dagger}_{\boldsymbol{i}\sigma}\hat{b}_{\boldsymbol{i}\sigma} + \hbar\omega_{eff}(\hat{a}^{\dagger}_{\boldsymbol{i}}\hat{a}_{\boldsymbol{i}} + \frac{1}{2}) + \sum_{\boldsymbol{j}}\sum_{int} J_{\boldsymbol{i}\sigma,\boldsymbol{j}\sigma',int}(\hat{b}^{\dagger}_{\boldsymbol{i}\sigma}\hat{b}_{\boldsymbol{j}\sigma'} + \hat{b}^{\dagger}_{\boldsymbol{j}\sigma'}\hat{b}_{\boldsymbol{i}}\sigma)\Big\} \tag{5}$$

Going to the reciprocal space the diagonal term simply remains diagonal:.

$$\sum_{\boldsymbol{n}\sigma} b^{\dagger}_{\boldsymbol{n}\sigma}b_{\boldsymbol{n}\sigma} = \sum_{\boldsymbol{k}} b^{\dagger}_{1,\boldsymbol{k}}b_{1,\boldsymbol{k}} + b^{\dagger}_{2,\boldsymbol{k}}b_{2,\boldsymbol{k}} \tag{6}$$

More delicate are the off-diagonal terms:

$$\sum_{\boldsymbol{n},\boldsymbol{m}}\sum_{\sigma,\sigma'} J_{\boldsymbol{n}\sigma,\boldsymbol{m}\sigma'}b^{\dagger}_{\boldsymbol{n}\sigma}b_{\boldsymbol{m}\sigma'} \tag{7}$$

where $\boldsymbol{n},\boldsymbol{m}$ run over the cells and σ,σ' run on the two molecules inside the unit cells. For the γ phase of quinacridone, using the vector $\boldsymbol{b} = (b,0)$ and $\boldsymbol{a} = (0,a)$ as the unit cell vectors, and recalling that there are two molecules per unit cell ($\sigma = \alpha,\beta$) the following interactions are defined:

$$J_{\boldsymbol{n}\sigma,\boldsymbol{m}\sigma'}\begin{cases} \sigma=\alpha: \begin{cases} \boldsymbol{m}=\boldsymbol{n}\pm\boldsymbol{b}, \sigma'=\alpha \xrightarrow{J_{\boldsymbol{n}\sigma,\boldsymbol{m}\sigma'}} J^{\gamma}_{\pi} \\ \begin{cases} \boldsymbol{m}=\boldsymbol{n}, \sigma'=\beta \xrightarrow{J_{\boldsymbol{n}\sigma,\boldsymbol{m}\sigma'}} J^{\gamma}_{H} \\ \boldsymbol{m}=\boldsymbol{n}-\boldsymbol{b}, \sigma'=\beta \xrightarrow{J_{\boldsymbol{n}\sigma,\boldsymbol{m}\sigma'}} J^{\gamma}_{H} \\ \boldsymbol{m}=\boldsymbol{n}-\boldsymbol{a}, \sigma'=\beta \xrightarrow{J_{\boldsymbol{n}\sigma,\boldsymbol{m}\sigma'}} J^{\gamma}_{H} \\ \boldsymbol{m}=\boldsymbol{n}-(\boldsymbol{a}+\boldsymbol{b}), \sigma'=\beta \xrightarrow{J_{\boldsymbol{n}\sigma,\boldsymbol{m}\sigma'}} J^{\gamma}_{H} \end{cases} \end{cases} \\ \sigma=\beta: \begin{cases} \boldsymbol{m}=\boldsymbol{n}\pm\boldsymbol{b}, \sigma'=\beta \xrightarrow{J_{\boldsymbol{n}\sigma,\boldsymbol{m}\sigma'}} J^{\gamma}_{\pi} \\ \begin{cases} \boldsymbol{m}=\boldsymbol{n}, \sigma'=\alpha \xrightarrow{J_{\boldsymbol{n}\sigma,\boldsymbol{m}\sigma'}} J^{\gamma}_{H} \\ \boldsymbol{m}=\boldsymbol{n}+\boldsymbol{b}, \sigma'=\alpha \xrightarrow{J_{\boldsymbol{n}\sigma,\boldsymbol{m}\sigma'}} J^{\gamma}_{H} \\ \boldsymbol{m}=\boldsymbol{n}+\boldsymbol{a}, \sigma'=\alpha \xrightarrow{J_{\boldsymbol{n}\sigma,\boldsymbol{m}\sigma'}} J^{\gamma}_{H} \\ \boldsymbol{m}=\boldsymbol{n}+(\boldsymbol{a}+\boldsymbol{b}), \sigma'=\alpha \xrightarrow{J_{\boldsymbol{n}\sigma,\boldsymbol{m}\sigma'}} J^{\gamma}_{H} \end{cases} \end{cases} \end{cases} \tag{8}$$

Looking at the interaction J_P that links two molecules of types σ,σ' located in the unit cells $\boldsymbol{n},\boldsymbol{m}=\boldsymbol{n}+\boldsymbol{s}$ we can use Eq 4 and generalize the interactions in Eq. 8 as:

$$\sum_{\boldsymbol{m},\boldsymbol{n}} J_P b^{\dagger}_{\boldsymbol{n},\sigma}b_{\boldsymbol{m},\sigma'} == \frac{J_P}{2}\sum_{\boldsymbol{k}\boldsymbol{s}} e^{i(\boldsymbol{r}_{\sigma}-\boldsymbol{r}_{\sigma'}-\boldsymbol{s})\boldsymbol{k}}(b^{\dagger}_{1,\boldsymbol{k}} \pm_{\sigma} b^{\dagger}_{2,\boldsymbol{k}})(b_{1,\boldsymbol{k}} \pm_{\sigma'} b_{2,\boldsymbol{k}}) \tag{9}$$

where with $\pm_{\sigma}$ I have indicated that the sign follows from the type of the molecule as in 4.

γ phase:J^{γ}_{π} terms

We apply 9 to the case in which $\sigma=\sigma'=\alpha$, as in Tab. 8.

$$J^{\gamma}_{\pi}\sum_{\boldsymbol{n}} b^{\dagger}_{\boldsymbol{n},\alpha}b_{\boldsymbol{n}+\boldsymbol{b},\alpha} + b^{\dagger}_{\boldsymbol{n},\alpha}b_{\boldsymbol{n}-\boldsymbol{b},\alpha} = \frac{J^{\gamma}_{\pi}}{2}[\sum_{\boldsymbol{k}} e^{-i\boldsymbol{b}\boldsymbol{k}}(b^{\dagger}_{1,\boldsymbol{k}} + b^{\dagger}_{2,\boldsymbol{k}})(b_{1,\boldsymbol{k}} + b_{2,\boldsymbol{k}}) + \sum_{\boldsymbol{k}} e^{i\boldsymbol{b}\boldsymbol{k}}(b^{\dagger}_{1,\boldsymbol{k}} + b^{\dagger}_{2,\boldsymbol{k}})(b_{1,\boldsymbol{k}} + b_{2,\boldsymbol{k}})] \tag{10}$$

Doing the same with $\sigma = \sigma' = \beta$ takes to:

$$\frac{J_{\pi}^{\gamma}}{2}\sum_{\boldsymbol{n}} b_{\boldsymbol{n},\beta}^{\dagger}b_{\boldsymbol{n}+\boldsymbol{b},\beta} + b_{\boldsymbol{n},\beta}^{\dagger}b_{\boldsymbol{n}-\boldsymbol{b},\beta} = \frac{J_{\pi}^{\gamma}}{2}[\sum_{\boldsymbol{k}} e^{-i\boldsymbol{b}\boldsymbol{k}}(b_{1,\boldsymbol{k}}^{\dagger} - b_{2,\boldsymbol{k}}^{\dagger})(b_{1,\boldsymbol{k}} - b_{2,\boldsymbol{k}}) + \sum_{\boldsymbol{k}} e^{i\boldsymbol{b}\boldsymbol{k}}(b_{1,\boldsymbol{k}}^{\dagger} - b_{2,\boldsymbol{k}}^{\dagger})(b_{1,\boldsymbol{k}} - b_{2,\boldsymbol{k}})] \tag{11}$$

Finally, if we sum together these two results we obtain:

$$\begin{aligned} &J_{\pi}^{\gamma}\sum_{\boldsymbol{n}} b_{\boldsymbol{n},\alpha}^{\dagger}b_{\boldsymbol{n}+\boldsymbol{b},\alpha} + b_{\boldsymbol{n},\alpha}^{\dagger}b_{\boldsymbol{n}-\boldsymbol{b},\alpha} + b_{\boldsymbol{n},\beta}^{\dagger}b_{\boldsymbol{n}+\boldsymbol{b},\beta} + b_{\boldsymbol{n},\beta}^{\dagger}b_{\boldsymbol{n}-\boldsymbol{b},\beta} = \\ &= 2J_{\pi}^{\gamma}\sum_{\boldsymbol{k}} cos(\boldsymbol{k}\boldsymbol{b})(b_{1,\boldsymbol{k}}^{\dagger}b_{1,\boldsymbol{k}} + b_{2,\boldsymbol{k}}^{\dagger}b_{2,\boldsymbol{k}}) \end{aligned} \tag{12}$$

All the cross terms cancel out and only the diagonal terms remain.

γ phase:J_H^{γ} terms

All terms have $\sigma \neq \sigma'$ and the phase exponentials do not expire. We then have 8 different terms:

$$J_H^{\gamma}\sum_{\boldsymbol{n}} b_{\boldsymbol{n},\alpha}^{\dagger}b_{\boldsymbol{n},\beta} = \frac{J_H^{\gamma}}{2}\sum_{\boldsymbol{k}} e^{i(\boldsymbol{r}_\beta - \boldsymbol{r}_\alpha - \boldsymbol{b} - \boldsymbol{a})\boldsymbol{k}}(b_{1,\boldsymbol{k}}^{\dagger} - b_{2,\boldsymbol{k}}^{\dagger})(b_{1,\boldsymbol{k}} + b_{2,\boldsymbol{k}}) \tag{13}$$

We expanded the relations in 8 using 9 The first thing to note is that, for the γ phase of QA, $\boldsymbol{r}_\beta - \boldsymbol{r}_\alpha = \frac{\boldsymbol{a}+\boldsymbol{b}}{2}$. So we can transform everything as:

$$J_H^{\gamma}\sum_{\boldsymbol{n}} b_{\boldsymbol{n},\alpha}^{\dagger}b_{\boldsymbol{n},\beta} = \frac{J_H^{\gamma}}{2}\sum_{\boldsymbol{k}} e^{-i(\frac{\boldsymbol{a}+\boldsymbol{b}}{2})\boldsymbol{k}}(b_{1,\boldsymbol{k}}^{\dagger} + b_{2,\boldsymbol{k}}^{\dagger})(b_{1,\boldsymbol{k}} - b_{2,\boldsymbol{k}}) \tag{14}$$

$$J_H^{\gamma}\sum_{\boldsymbol{n}} b_{\boldsymbol{n},\alpha}^{\dagger}b_{\boldsymbol{n}-\boldsymbol{b},\beta} = \frac{J_H^{\gamma}}{2}\sum_{\boldsymbol{k}} e^{-i(\frac{\boldsymbol{a}-\boldsymbol{b}}{2})\boldsymbol{k}}(b_{1,\boldsymbol{k}}^{\dagger} + b_{2,\boldsymbol{k}}^{\dagger})(b_{1,\boldsymbol{k}} - b_{2,\boldsymbol{k}}) \tag{15}$$

$$J_H^{\gamma}\sum_{\boldsymbol{n}} b_{\boldsymbol{n},\alpha}^{\dagger}b_{\boldsymbol{n}-\boldsymbol{a},\beta} = \frac{J_H^{\gamma}}{2}\sum_{\boldsymbol{k}} e^{-i(\frac{-\boldsymbol{a}+\boldsymbol{b}}{2})\boldsymbol{k}}(b_{1,\boldsymbol{k}}^{\dagger} + b_{2,\boldsymbol{k}}^{\dagger})(b_{1,\boldsymbol{k}} - b_{2,\boldsymbol{k}}) \tag{16}$$

$$J_H^{\gamma}\sum_{\boldsymbol{n}} b_{\boldsymbol{n},\alpha}^{\dagger}b_{\boldsymbol{n}-\boldsymbol{a}-\boldsymbol{b},\beta} = \frac{J_H^{\gamma}}{2}\sum_{\boldsymbol{k}} e^{-i(\frac{-\boldsymbol{a}-\boldsymbol{b}}{2})\boldsymbol{k}}(b_{1,\boldsymbol{k}}^{\dagger} + b_{2,\boldsymbol{k}}^{\dagger})(b_{1,\boldsymbol{k}} - b_{2,\boldsymbol{k}}) \tag{17}$$

$$J_H^{\gamma}\sum_{\boldsymbol{n}} b_{\boldsymbol{n},\beta}^{\dagger}b_{\boldsymbol{n},\alpha} = \frac{J_H^{\gamma}}{2}\sum_{\boldsymbol{k}} e^{i(\frac{\boldsymbol{a}+\boldsymbol{b}}{2})\boldsymbol{k}}(b_{1,\boldsymbol{k}}^{\dagger} - b_{2,\boldsymbol{k}}^{\dagger})(b_{1,\boldsymbol{k}} + b_{2,\boldsymbol{k}}) \tag{18}$$

$$J_H^{\gamma}\sum_{\boldsymbol{n}} b_{\boldsymbol{n},\beta}^{\dagger}b_{\boldsymbol{n}+\boldsymbol{b},\alpha} = \frac{J_H^{\gamma}}{2}\sum_{\boldsymbol{k}} e^{i(\frac{\boldsymbol{a}-\boldsymbol{b}}{2})\boldsymbol{k}}(b_{1,\boldsymbol{k}}^{\dagger} - b_{2,\boldsymbol{k}}^{\dagger})(b_{1,\boldsymbol{k}} + b_{2,\boldsymbol{k}}) \tag{19}$$

$$J_H^{\gamma}\sum_{\boldsymbol{n}} b_{\boldsymbol{n},\beta}^{\dagger}b_{\boldsymbol{n}+\boldsymbol{a},\alpha} = \frac{J_H^{\gamma}}{2}\sum_{\boldsymbol{k}} e^{i(\frac{-\boldsymbol{a}+\boldsymbol{b}}{2})\boldsymbol{k}}(b_{1,\boldsymbol{k}}^{\dagger} - b_{2,\boldsymbol{k}}^{\dagger})(b_{1,\boldsymbol{k}} + b_{2,\boldsymbol{k}}) \tag{20}$$

$$J_H^{\gamma}\sum_{\boldsymbol{n}} b_{\boldsymbol{n},\beta}^{\dagger}b_{\boldsymbol{n}+\boldsymbol{b}+\boldsymbol{a},\alpha} = \frac{J_H^{\gamma}}{2}\sum_{\boldsymbol{k}} e^{i(\frac{-\boldsymbol{a}-\boldsymbol{b}}{2})\boldsymbol{k}}(b_{1,\boldsymbol{k}}^{\dagger} - b_{2,\boldsymbol{k}}^{\dagger})(b_{1,\boldsymbol{k}} + b_{2,\boldsymbol{k}}) \tag{21}$$

we can now group the terms in couples:

$$\begin{aligned} \boldsymbol{s} = \boldsymbol{0} : \frac{J_H^{\gamma}}{2}\sum_{\boldsymbol{k}} e^{-i(\frac{\boldsymbol{a}+\boldsymbol{b}}{2})\boldsymbol{k}}(b_{1,\boldsymbol{k}}^{\dagger} + b_{2,\boldsymbol{k}}^{\dagger})(b_{1,\boldsymbol{k}} - b_{2,\boldsymbol{k}}) + e^{i(\frac{\boldsymbol{a}+\boldsymbol{b}}{2})\boldsymbol{k}}(b_{1,\boldsymbol{k}}^{\dagger} - b_{2,\boldsymbol{k}}^{\dagger})(b_{1,\boldsymbol{k}} + b_{2,\boldsymbol{k}}) \\ \boldsymbol{s} = \pm\boldsymbol{b} : \frac{J_H^{\gamma}}{2}\sum_{\boldsymbol{k}} e^{-i(\frac{\boldsymbol{a}-\boldsymbol{b}}{2})\boldsymbol{k}}(b_{1,\boldsymbol{k}}^{\dagger} + b_{2,\boldsymbol{k}}^{\dagger})(b_{1,\boldsymbol{k}} - b_{2,\boldsymbol{k}}) + e^{i(\frac{\boldsymbol{a}-\boldsymbol{b}}{2})\boldsymbol{k}}(b_{1,\boldsymbol{k}}^{\dagger} - b_{2,\boldsymbol{k}}^{\dagger})(b_{1,\boldsymbol{k}} + b_{2,\boldsymbol{k}}) \\ \boldsymbol{s} = \pm\boldsymbol{a} : \frac{J_H^{\gamma}}{2}\sum_{\boldsymbol{k}} e^{-i(\frac{-\boldsymbol{a}+\boldsymbol{b}}{2})\boldsymbol{k}}(b_{1,\boldsymbol{k}}^{\dagger} + b_{2,\boldsymbol{k}}^{\dagger})(b_{1,\boldsymbol{k}} - b_{2,\boldsymbol{k}}) + e^{i(\frac{-\boldsymbol{a}+\boldsymbol{b}}{2})\boldsymbol{k}}(b_{1,\boldsymbol{k}}^{\dagger} - b_{2,\boldsymbol{k}}^{\dagger})(b_{1,\boldsymbol{k}} + b_{2,\boldsymbol{k}}) \\ \boldsymbol{s} = \pm(\boldsymbol{a}+\boldsymbol{b}) : \frac{J_H^{\gamma}}{2}\sum_{\boldsymbol{k}} e^{-i(\frac{-\boldsymbol{a}-\boldsymbol{b}}{2})\boldsymbol{k}}(b_{1,\boldsymbol{k}}^{\dagger} + b_{2,\boldsymbol{k}}^{\dagger})(b_{1,\boldsymbol{k}} - b_{2,\boldsymbol{k}}) + e^{i(\frac{-\boldsymbol{a}-\boldsymbol{b}}{2})\boldsymbol{k}}(b_{1,\boldsymbol{k}}^{\dagger} - b_{2,\boldsymbol{k}}^{\dagger})(b_{1,\boldsymbol{k}} + b_{2,\boldsymbol{k}}) \end{aligned}$$

expanding the products and the exponentials with the Euler formula and summing everything takes to:

$$\begin{aligned} \frac{J_H^{\gamma}}{2}\sum_{\boldsymbol{k}}\{&2cos\Big[\boldsymbol{k}\frac{(\boldsymbol{a}+\boldsymbol{b})}{2}\Big(b_{1,\boldsymbol{k}}^{\dagger}b_{1,\boldsymbol{k}} - b_{2,\boldsymbol{k}}^{\dagger}b_{2,\boldsymbol{k}}) - 2isin\Big[\boldsymbol{k}\frac{(\boldsymbol{a}+\boldsymbol{b})}{2}\Big](b_{1,\boldsymbol{k}}^{\dagger}b_{2,\boldsymbol{k}} - b_{2,\boldsymbol{k}}^{\dagger}b_{1,\boldsymbol{k}}) + \\ &+2cos\Big[\boldsymbol{k}\frac{(\boldsymbol{a}-\boldsymbol{b})}{2}\Big](b_{1,\boldsymbol{k}}^{\dagger}b_{1,\boldsymbol{k}} - b_{2,\boldsymbol{k}}^{\dagger}b_{2,\boldsymbol{k}}) - 2isin\Big[\boldsymbol{k}\frac{(\boldsymbol{a}-\boldsymbol{b})}{2}\Big](b_{1,\boldsymbol{k}}^{\dagger}b_{2,\boldsymbol{k}} - b_{2,\boldsymbol{k}}^{\dagger}b_{1,\boldsymbol{k}}) + \\ &+2cos\Big[\boldsymbol{k}\frac{(-\boldsymbol{a}+\boldsymbol{b})}{2}\Big](b_{1,\boldsymbol{k}}^{\dagger}b_{1,\boldsymbol{k}} - b_{2,\boldsymbol{k}}^{\dagger}b_{2,\boldsymbol{k}}) - 2isin\Big[\boldsymbol{k}\frac{(-\boldsymbol{a}+\boldsymbol{b})}{2}\Big](b_{1,\boldsymbol{k}}^{\dagger}b_{2,\boldsymbol{k}} - b_{2,\boldsymbol{k}}^{\dagger}b_{1,\boldsymbol{k}}) + \\ &+2cos\Big[\boldsymbol{k}\frac{(-\boldsymbol{a}-\boldsymbol{b})}{2}\Big](b_{1,\boldsymbol{k}}^{\dagger}b_{1,\boldsymbol{k}} - b_{2,\boldsymbol{k}}^{\dagger}b_{2,\boldsymbol{k}}) - 2isin\Big[\boldsymbol{k}\frac{(-\boldsymbol{a}-\boldsymbol{b})}{2}\Big](b_{1,\boldsymbol{k}}^{\dagger}b_{2,\boldsymbol{k}} - b_{2,\boldsymbol{k}}^{\dagger}b_{1,\boldsymbol{k}})\} \end{aligned}$$

Finally, we use the fact that the sine is an odd function while the cosine is odd. The cosine terms with opposite sign do sum up, while the sine terms cancel each other, so that:

$$2J_H^\gamma \sum_{\boldsymbol{k}} \{cos\Big[\boldsymbol{k}\frac{(\boldsymbol{a}+\boldsymbol{b})}{2}\Big](b_{1,\boldsymbol{k}}^\dagger b_{1,\boldsymbol{k}} - b_{2,\boldsymbol{k}}^\dagger b_{2,\boldsymbol{k}}) + cos\Big[\boldsymbol{k}\frac{(\boldsymbol{a}-\boldsymbol{b})}{2}\Big](b_{1,\boldsymbol{k}}^\dagger b_{1,\boldsymbol{k}} - b_{2,\boldsymbol{k}}^\dagger b_{2,\boldsymbol{k}})\}$$

Finally, the complete electronic exciton Hamiltonian in the reciprocal space reads:

$$\begin{aligned}\hat{H}_{el} = \sum_{\boldsymbol{k}} \Big\{ &\{\epsilon_0 + D + 2J_\pi^\gamma cos(\boldsymbol{kb}) + 2J_H^\gamma cos[\boldsymbol{k}(\frac{\boldsymbol{a}+\boldsymbol{b}}{2})] + 2J_H^\gamma cos[\boldsymbol{k}(\frac{\boldsymbol{a}-\boldsymbol{b}}{2})]\} b_{1,\boldsymbol{k}}^\dagger b_{1,\boldsymbol{k}} + \\ &+ \{\epsilon_0 + D + 2J_\pi^\gamma cos(\boldsymbol{kb}) - 2J_H^\gamma cos[\boldsymbol{k}(\frac{\boldsymbol{a}+\boldsymbol{b}}{2})] - 2J_H^\gamma cos[\boldsymbol{k}(\frac{\boldsymbol{a}-\boldsymbol{b}}{2})]\} b_{2,\boldsymbol{k}}^\dagger b_{2,\boldsymbol{k}} \Big\}\end{aligned} \tag{22}$$

Vibrations

A similar procedure is adopted to deal with vibrations.

$$a_{\boldsymbol{n}\alpha}^\dagger = \frac{1}{\sqrt{2N}} \sum_{\boldsymbol{Q}} e^{i\boldsymbol{Q}(\boldsymbol{n}+\boldsymbol{r}_\alpha)}(a_{1,\boldsymbol{Q}}^\dagger + a_{2,\boldsymbol{Q}}^\dagger) \tag{23}$$

$$a_{\boldsymbol{n}\beta}^\dagger = \frac{1}{\sqrt{2N}} \sum_{\boldsymbol{Q}} e^{i\boldsymbol{Q}(\boldsymbol{n}+\boldsymbol{r}_\beta)}(a_{1,\boldsymbol{Q}}^\dagger - a_{2,\boldsymbol{Q}}^\dagger) \tag{24}$$

Exactly as the electronic Hamiltonian, the diagonal term of the vibrations remains diagonal:

$$\sum_{\boldsymbol{n},\sigma} a_{\boldsymbol{n},\sigma}^\dagger a_{\boldsymbol{n},\sigma} = \sum_{\boldsymbol{Q}} a_{1,\boldsymbol{Q}}^\dagger a_{1,\boldsymbol{Q}} + a_{2,\boldsymbol{Q}}^\dagger a_{2,\boldsymbol{Q}} \tag{25}$$

We can then work out the vibronic coupling term:

$$\begin{aligned}&g\sum_{\boldsymbol{n},\sigma}(a_{\boldsymbol{n},\sigma}^\dagger + a_{\boldsymbol{n},\sigma}) b_{\boldsymbol{n},\sigma}^\dagger b_{\boldsymbol{n},\sigma} = \\ &+\frac{g}{\sqrt{2N}}\sum_{\boldsymbol{k},\boldsymbol{Q}}\{a_{1,\boldsymbol{Q}}^\dagger b_{1,\boldsymbol{k}}^\dagger b_{1,\boldsymbol{k}+\boldsymbol{Q}} + a_{1,\boldsymbol{Q}}^\dagger b_{2,\boldsymbol{k}}^\dagger b_{2,\boldsymbol{k}+\boldsymbol{Q}} + a_{2,\boldsymbol{Q}}^\dagger b_{1,\boldsymbol{k}}^\dagger b_{2,\boldsymbol{k}+\boldsymbol{Q}} + a_{2,\boldsymbol{Q}}^\dagger b_{2,\boldsymbol{k}}^\dagger b_{1,\boldsymbol{k}+\boldsymbol{Q}} + h.c.\}\end{aligned} \tag{26}$$

Only the terms with an even number of creation/annihilation operators concerning the second branches survive. The final Hamiltonian reads:

$$\begin{aligned}\hat{H} = \sum_{\boldsymbol{k}} \Big\{ &\{\epsilon_0 + D + 2J_\pi^\gamma cos(\boldsymbol{kb}) + 2J_H^\gamma cos[\boldsymbol{k}(\frac{\boldsymbol{a}+\boldsymbol{b}}{2})] + 2J_H^\gamma cos[\boldsymbol{k}(\frac{\boldsymbol{a}-\boldsymbol{b}}{2})]\} b_{1,\boldsymbol{k}}^\dagger b_{1,\boldsymbol{k}} + \\ &+ \{\epsilon_0 + D + 2J_\pi^\gamma cos(\boldsymbol{kb}) - 2J_H^\gamma cos[\boldsymbol{k}(\frac{\boldsymbol{a}+\boldsymbol{b}}{2})] - 2J_H^\gamma cos[\boldsymbol{k}(\frac{\boldsymbol{a}-\boldsymbol{b}}{2})]\} b_{2,\boldsymbol{k}}^\dagger b_{2,\boldsymbol{k}} \Big\} + \\ &+ h\omega \sum_{\boldsymbol{Q}} (a_{1,\boldsymbol{Q}}^\dagger a_{1,\boldsymbol{Q}} + a_{2,\boldsymbol{Q}}^\dagger a_{2,\boldsymbol{Q}}) + \\ &+ \frac{g}{\sqrt{2N}}\sum_{\boldsymbol{k},\boldsymbol{Q}}\Big\{a_{1,\boldsymbol{Q}}^\dagger b_{1,\boldsymbol{k}}^\dagger b_{1,\boldsymbol{k}+\boldsymbol{Q}} + a_{1,\boldsymbol{Q}}^\dagger b_{2,\boldsymbol{k}}^\dagger b_{2,\boldsymbol{k}+\boldsymbol{Q}} + a_{2,\boldsymbol{Q}}^\dagger b_{1,\boldsymbol{k}}^\dagger b_{2,\boldsymbol{k}+\boldsymbol{Q}} + a_{2,\boldsymbol{Q}}^\dagger b_{2,\boldsymbol{k}}^\dagger b_{1,\boldsymbol{k}+\boldsymbol{Q}} + h.c.\Big\}\end{aligned} \tag{27}$$

where J_π^γ, J_H^γ are the π/hydrogen bond interactions, $\boldsymbol{a}, \boldsymbol{b}$ are the unit cell vector, $b_{i,\boldsymbol{k}}^\dagger/b_{i,\boldsymbol{k}}$ creates/annihilates an exciton on the i branch at wavevector $\boldsymbol{k}$, $a_{i,\boldsymbol{Q}}^\dagger/a_{i,\boldsymbol{Q}}$ crates/annihilates a phonon on the i branch at wavevector $\boldsymbol{Q}$.

Dipole moment operator

$$\begin{aligned}\hat{\boldsymbol{\mu}} &= \sum_{\boldsymbol{n}\sigma} \boldsymbol{\mu}_{\boldsymbol{n}\sigma}(b_{\boldsymbol{n}\sigma}^\dagger + b_{\boldsymbol{n}\sigma}) = \sum_{\boldsymbol{n}} \boldsymbol{\mu}_\alpha(b_{\boldsymbol{n}\alpha}^\dagger + b_{\boldsymbol{n}\alpha}) + \boldsymbol{\mu}_\beta(b_{\boldsymbol{n}\beta}^\dagger + b_{\boldsymbol{n}\beta}) = \\ &= \sqrt{N}[(\boldsymbol{\mu}_\alpha + \boldsymbol{\mu}_\beta)(b_{1,\boldsymbol{k}=0}^\dagger + b_{1,\boldsymbol{k}=0}) + (\boldsymbol{\mu}_\alpha - \boldsymbol{\mu}_\beta)(b_{2,\boldsymbol{k}=0}^\dagger + b_{2,\boldsymbol{k}=0})]\end{aligned} \tag{28}$$

5 Simulation of absorption and emission spectra

Absorption and emission spectra are calculated assigning a Gaussian lineshape to each transition:

$$I_{abs}(\omega) \propto \frac{\hbar\omega}{\sigma} \sum_{f>i} |\langle f| \hat{\mu} |i\rangle|^2 e^{-\frac{\hbar^2}{2\sigma^2}(\omega_{fi}-\omega)^2} \tag{29}$$

$$I_{emi}(\omega) \propto \frac{(\hbar\omega)^3}{\sigma} \sum_{f<i} |\langle f| \hat{\mu} |i\rangle|^2 e^{-\frac{\hbar^2}{2\sigma^2}(\omega_{fi}-\omega)^2} \tag{30}$$

where I is the intensity of the spectra at frequency ω, σ is the Gaussian width, $\hbar\omega_{if}$ is the energy of the transition between states i and f. In absorption (emission) experiments, f runs on all states with energy higher (lower) than the ground (Kasha) state.For an easier comparison with experiments, all spectra are normalized.

6 Emission and absorption spectra

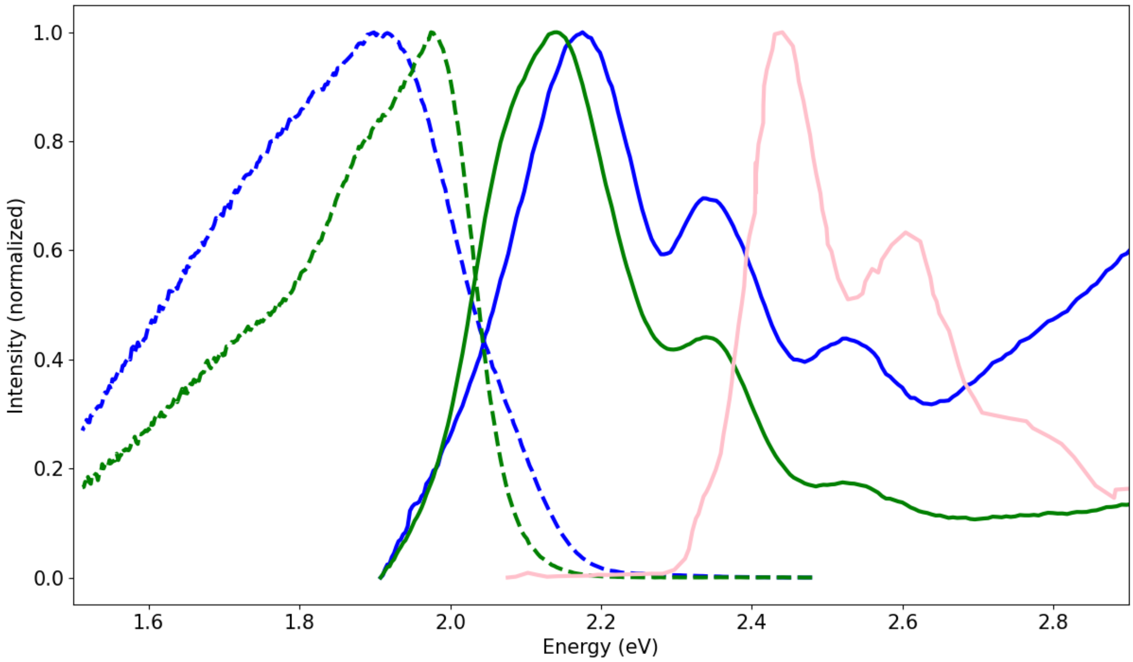

Figure S18: **Experimental spectra.** Experimental absorption (continuous lines) and emission (dashed lines) spectra of βQA (green), γQA (blue) (obtained in this work). Pink lines show the absorption spectrum of QA in dioxane solution from literature.[2]

	βQA		γQA	
	J_H	J_π	J_H	J_π
This work	-0.013	0.008	-0.008	0.020
Dipole approximation	-0.012	0.036	-0.016	0.011
This work (no refractive index correction)	-0.052	0.033	-0.031	0.078

Table S20: **Comparison between possible choices for the excitonic couplings.** Excitonic couplings calculated following different methods for βQA and γQA . Up: fitting with TDDFT and dividing by the squared refractive index as discussed in the main text. Middle: point-dipole approximation (again accounting for the refractive index correction). Bottom: as the top line but without accounting for the refractive index correction.

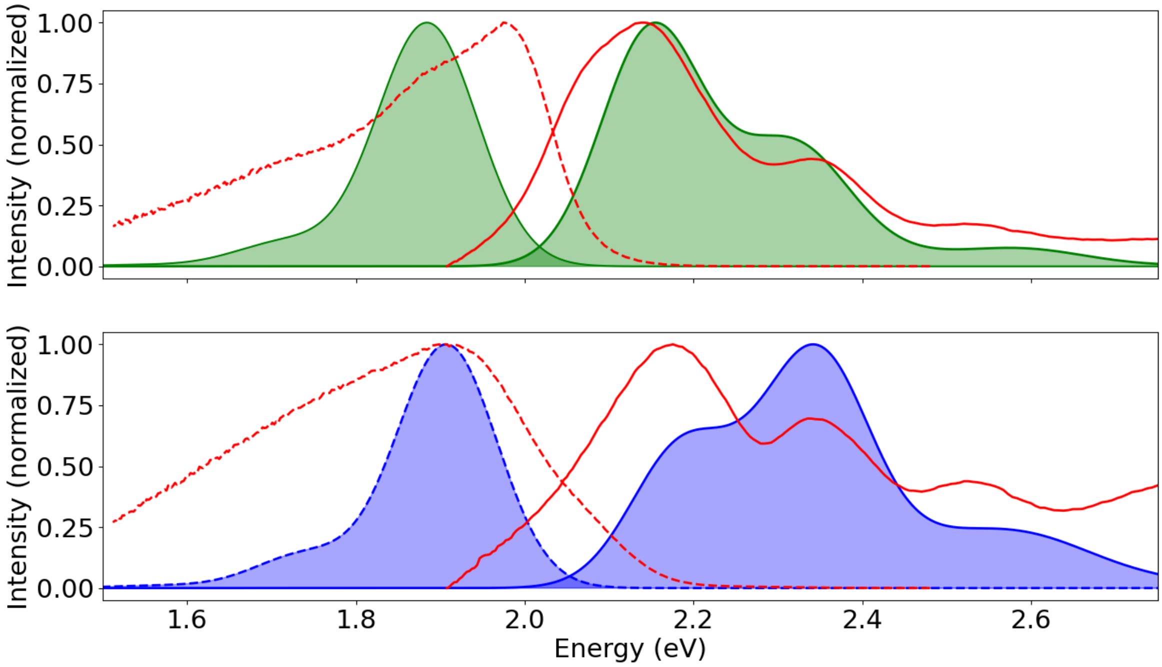

Figure S19: **QA spectra using Js without refractive index correction.** Calculated absorption and emission spectra of QA β (top) and γQA (bottom) of QA using directly the fitted J (without correction for the squared refractive index), compared with experimental measurements (red lines). Calculated spectra are shown in green(βQA) and blue (γQA). Solid lines are used for experimental absorption spectra and dashed lines for experimental emission spectra.

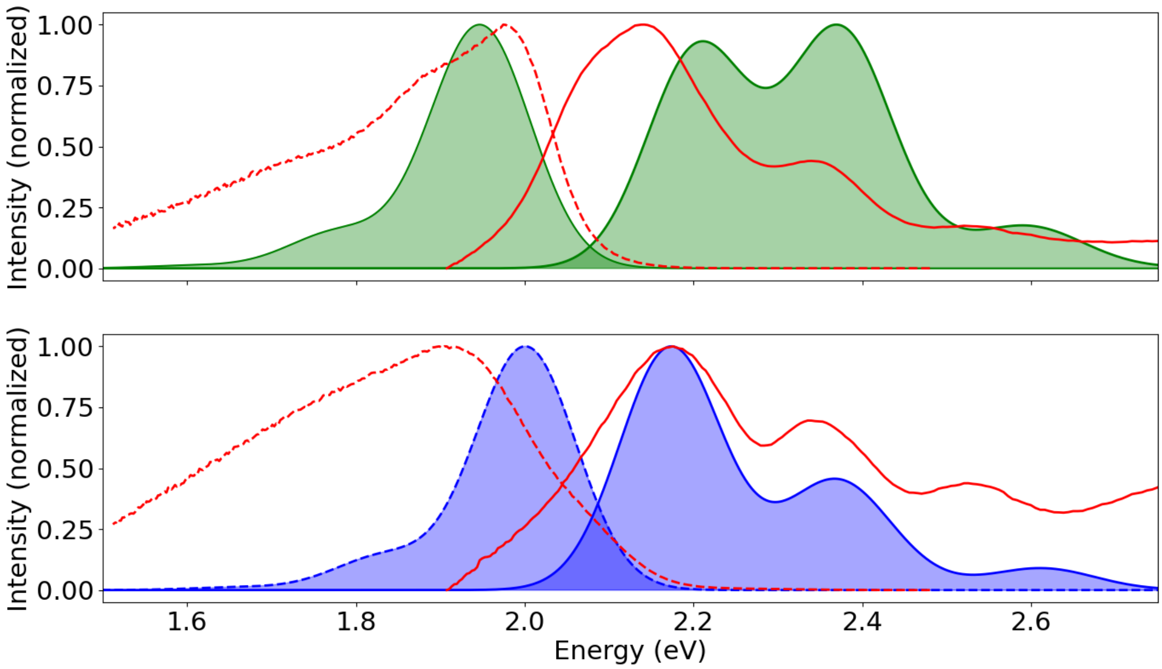

Figure S20: **QA spectra using Js with the point dipole approximation.** Calculated absorption and emission spectra of QA β (top) and γQA (bottom) of QA using the J calculated with the dipole moment approximation, compared with experimental measurements (red lines). Calculated spectra shown in green(βQA) and blue (γQA). Solid lines are used for experimental absorption spectra and dashed lines for experimental emission spectra.

References

[1] Erich F Paulus, Frank JJ Leusen, and Martin U Schmidt. Crystal structures of quinacridones. *CrystEngComm*, 9(2):131–143, 2007.

[2] Jin Mizuguchi and Takatoshi Senju. Solution and solid-state spectra of quinacridone derivatives as viewed from the intermolecular hydrogen bond. *The Journal of Physical Chemistry B*, 110(39):19154–19161, 2006.

[3] Felix Plasser. Theodore: A toolbox for a detailed and automated analysis of electronic excited state computations. *The Journal of Chemical Physics*, 152(8):084108, 2020.

[4] Pauli Virtanen, Ralf Gommers, Travis E. Oliphant, Matt Haberland, Tyler Reddy, David Cournapeau, Evgeni Burovski, Pearu Peterson, Warren Weckesser, Jonathan Bright, Stéfan J. van der Walt, Matthew Brett, Joshua Wilson, K. Jarrod Millman, Nikolay Mayorov, Andrew R. J. Nelson, Eric Jones, Robert Kern, Eric Larson, C J Carey, İlhan Polat, Yu Feng, Eric W. Moore, Jake VanderPlas, Denis Laxalde, Josef Perktold, Robert Cimrman, Ian Henriksen, E. A. Quintero, Charles R. Harris, Anne M. Archibald, Antônio H. Ribeiro, Fabian Pedregosa, Paul van Mulbregt, and SciPy 1.0 Contributors. SciPy 1.0: Fundamental Algorithms for Scientific Computing in Python. *Nature Methods*, 17:261–272, 2020.

[5] M Anzola, F Di Maiolo, and A Painelli. Optical spectra of molecular aggregates and crystals: testing approximation schemes. *Physical Chemistry Chemical Physics*, 21(36):19816–19824, 2019.

[6] Mattia Anzola and Anna Painelli. Aggregates of polar dyes: Beyond the exciton model. *Physical Chemistry Chemical Physics*, 23(14):8282–8291, 2021.

[7] Vladimir M Agranovich. *Excitations in organic solids*, volume 142. OUP Oxford, 2009.
